# The plant growth-promoting *Burkholderia vietnamiensis* produces acyl-homoserine lactones and modulates the quorum-sensing signaling in the rhizosphere

**DOI:** 10.3389/fmicb.2025.1638793

**Published:** 2025-08-15

**Authors:** Selvaraj Poonguzhali, Kiyoon Kim, Munusamy Madhaiyan, Tongmin Sa

**Affiliations:** ^1^Department of Environmental and Biological Chemistry, Chungbuk National University, Cheongju, Republic of Korea; ^2^Temasek Life Sciences Laboratory (TLL), National University of Singapore (NUS), Singapore, Singapore; ^3^National Forest Seed Variety Center, Chungju, Republic of Korea; ^4^Singapore Institute of Food and Biotechnology Innovation (SIFBI), Agency for Science, Technology and Research (A*STAR), Singapore, Singapore; ^5^The Korean Academy of Science and Technology, Seongnam, Republic of Korea

**Keywords:** quorum sensing, *N*-acyl homoserine lactones, *Burkholderia vietnamiensis*, rhizosphere, PGPR, biocontrol

## Abstract

The genus *Burkholderia*, comprising over 60 species, represents a highly diverse group of bacteria known for their exceptional metabolic versatility. Quorum sensing (QS), a mechanism of cell-density-dependent gene regulation, plays a critical role in host colonization, environmental adaptation, and, in many cases, pathogenesis. Due to the established link between QS and virulence, most QS studies in *Burkholderia cepacia* complex (Bcc) species have focused on pathogenic strains. In contrast, comparatively little attention has been given to QS in plant growth-promoting (PGP) *Burkholderia* strains. In this study, we investigated the QS systems of *Burkholderia vietnamiensis* strains with plant growth-promoting potential. We identified two functional QS circuits, CepI/R and BviI/R, responsible for the synthesis of distinct AHL molecules with *N*-decanoyl homoserine lactone (C_10_-HSL) as the dominant molecule. In *B. vietnamiensis* CBMB40, both synthases contributed to the production of N-hexanoyl (C_6_-) and *N*-Octanoyl (C_8_-) homoserine lactones, while *bviI* synthase contributed to the production of C_10_-HSL and *N*-Dodecanoyl (C_12_-) homoserine lactones. The AHLs produced by CBMB40 could be detected in plant tissues, and they served as interpopulation signaling molecules within the rhizosphere. A random transposon mutagenesis approach was employed to generate an AHL-deficient mutant (ΔCBMB40). The mutant exhibited an extended log phase, reduced protease activity, and loss of antagonism against *Erwinia carotovora* subsp. *carotovora*, as well as diminished activity against multiple fungal pathogens. Notably, the addition of AHL extracts from the wild-type strain restored antagonistic activity in the mutant. Furthermore, *in vitro* potato tuber assays and pot culture experiments in red pepper confirmed that AHL-mediated QS is essential for the biocontrol potential of CBMB40. Together, these findings enhance our understanding of QS-regulated functions in PGP *B. vietnamiensis* CBMB40 and support its potential application as a sustainable biocontrol agent in agriculture. Importantly, this study underscores the potential of using PGP bacteria (PGPB) to prime plant defenses, offering a biologically meaningful and ecologically sustainable alternative to genetically modified plants engineered with AHL synthase genes. AHL-mediated cross-communication in the rhizosphere may further disrupt pathogenic signaling, opening new avenues for microbiome-based crop protection strategies.

## 1 Introduction

The rhizosphere is a dynamic region of soil surrounding plant roots where plant–microbe associations are shaped by intricate interactions and feedback mechanisms involving roots, microorganisms, and the soil environment (Zhalnina et al., 2018). These interactions are largely governed by a process known as chemo-signaling, wherein a variety of small molecules and metabolites act as mediators for both plant–microbe and microbe–microbe communication, triggering specific responses in plants (Chagas et al., 2018; Santoyo, 2022). In recent years, quorum sensing (QS) has gained increasing recognition as a key component of chemical communication and a crucial mediator of plant–microbe interactions in the rhizosphere. QS is a regulatory mechanism that enables bacteria to coordinate gene expression in a population density-dependent manner and communicate both with other microbes and with host organisms (prokaryotic and eukaryotic) through the release of signaling molecules known as autoinducers. An emerging perspective suggests that bacteria utilize QS as a strategy for collective environmental sensing through social interactions (Moreno-Gámez et al., 2023). As such, QS facilitates both intra- and interspecies microbial communication, as well as interaction with host organisms, even at the level of single bacterial cells. In Gram-negative bacteria, including plant growth-promoting rhizobacteria (PGPR), a group known for their positive effects on plant growth and health, the autoinducers are primarily *N*-acyl-homoserine lactones (AHLs). These molecules are synthesized by specific bacterial enzymes. They are composed of a conserved homoserine lactone ring linked via an amide bond to an acyl side chain that can vary in length (C_4_ to C_18_), the acyl group either saturated or unsaturated, and substituted at the third carbon position (C_3_) with either an oxo or hydroxyl group.

In PGPR, AHL-mediated QS regulates critical traits such as biofilm formation, root colonization, biocontrol activity, production of antimicrobial compounds, and the synthesis of phytohormones. Accumulating evidence indicates that these signal molecules, particularly AHLs, are not solely confined to bacterial communication but are also recognized by plants, which often exhibit specific physiological and molecular responses to these compounds (Mathesius et al., 2003; Soto et al., 2006). AHLs have been shown to influence various aspects of plant development and stress response, including modulation of gene expression, root architecture, plant defense activation, and the regulation of metabolic and hormonal pathways (Zhuang et al., 2023). Importantly, the nature of these effects is highly dependent on the type of AHL molecule, particularly the length and chemical modifications of the acyl side chain. In most cases, it is either linked to enhanced growth rates and elongation of the primary root via auxins modulation or induction of resistance and priming of plant defense (Venturi and Keel, 2016; Pazarlar et al., 2020; Ibal et al., 2021; Gahoi et al., 2021; Duan et al., 2024). Interestingly, plants can also be bioengineered to produce bacterial AHLs by expressing bacterial AHL synthase genes, and the produced AHLs are capable of modulating microbial interactions, potentially promoting beneficial relationships or deterring pathogenic ones (Fray et al., 1999; Mäe et al., 2001; Toth et al., 2004). These engineered plants can exude AHLs from both roots and leaf surfaces, allowing the signaling molecules to diffuse into the rhizosphere and even into the bulk soil beyond the root zone (Scott et al., 2006). However, this approach is not without potential drawbacks. Since plants naturally produce AHL mimics as part of their defense strategy, engineering them to synthesize authentic AHLs might interfere with innate defense mechanisms and might render them more susceptible to infection (Fray, 2002). Additionally, AHL signaling plays a critical role in regulating colonization and behavior of many plant-associated beneficial microbes, and there is evidence for cross-communication via AHLs among rhizospheric bacterial populations (Pierson et al., 1998; Steidle et al., 2002; Fray, 2002). Therefore, disrupting native AHL-mediated communication networks might inadvertently hinder colonization or activity of key growth-promoting or biocontrol bacteria (Zhang and Pierson, 2001). Hence, it is worth emphasizing that PGPR offers a promising alternative to the genetic engineering of plants with AHLs. It can be hypothesized that priming plants with AHLs naturally produced by PGPR or other endophytic plant growth-promoting bacteria (PGPB) may confer similar resistance benefits, provided these signaling molecules are present within plant tissues. Moreover, since PGPB are natural root colonizers, their use is less likely to disrupt native microbial communities or interfere with existing signaling networks. This approach minimizes the risk of altering beneficial plant–microbe interactions and maintains the ecological balance in the rhizosphere. In light of the points discussed above, we previously showed that the AHLs produced by endophytic PGPB strains from *Burkholderia* can be detected *in planta* via TLC coupled to bioassays with the indicator strains (Poonguzhali et al., 2007). In this study, we show that *Burkholderia vietnamiensis* strains produce a mixture of AHLs using Gas Chromatography Mass spectrometry (GC–MS) analysis and further prove that *B. vietnamiensis* CBMB40 produces AHLs *in situ* in the rhizosphere, which is used for intercommunication signaling and primes the plant defence against the soft rot pathogens. The genus *Burkholderia*, belonging to the class *β-Proteobacteria,* is a genetically diverse group comprising over 60 species known for their remarkable metabolic versatility. Taxonomic analysis of *Burkholderia* reveals a division into two major clusters. The first includes pathogenic species, such as *Burkholderia mallei* and members of the *Burkholderia cepacia* complex (Bcc), known for their clinical relevance. The second, more recently defined cluster consists of non-pathogenic environmental species, many of which are associated with plants. These plant-associated species exhibit several beneficial traits, including rhizosphere and endophytic colonization, plant growth promotion, nitrogen fixation, phosphate solubilization, degradation of aromatic compounds, and symbiotic associations with plants and mosses (Suárez-Moreno et al., 2010).

Quorum sensing has been extensively studied in various *Burkholderia* species, and a range of AHL molecules, medium- to long-chain variants (C_6_-HSL to C_14_-HSL), with or without substitutions, have been reported. These AHLs regulate traits, such as protease production, biofilm formation, motility, and synthesis of bioactive compounds. In the Bcc, a conserved CepI/CepR QS system is widely present, which specifically synthesizes and responds to *N*-hexanoyl (C_6_-) and *N*-octanoyl homoserine lactones (C_8_-HSL). Several other *Burkholderia* species possess multiple LuxI/R-type QS systems, often linked to the regulation of virulence-associated traits. In contrast, QS in non-pathogenic, nitrogen-fixing *Burkholderia* strains associated with plants remains comparatively less understood. For instance, *Burkholderia kururiensis* and PGPB within this cluster carry BraI/R-like QS systems, while *B. xenovorans* harbors a unique and poorly characterized XenI2/R2 QS circuit. These plant-associated species display both conserved and distinct QS features, likely shaped by ecological adaptations to their environmental niches (Suárez-Moreno et al., 2010).

Quorum sensing in *B. vietnamiensis*, a species mainly represented by environmental isolates, involves production of long-chain AHLs. Among these, N-decanoyl-homoserine lactone (C_10_-HSL) is the most abundant signal molecule identified. In addition to the CepI/CepR system, environmental strains of *B. vietnamiensis* possess a second QS circuit, BviI/R, which specifically governs the synthesis of C_10_-HSL. Notably, C_10_-HSL has not been detected in clinical isolates, and the specific functional roles of this molecule, as well as the BviI/R system, remain largely unexplored (Gotschlich et al., 2001; Conway and Greenberg, 2002; Malott and Sokol, 2007).

Our previous studies demonstrated that *B. vietnamiensis* and other PGP *Burkholderia* sp. produce a diverse range of AHLs, which can also be detected under *in planta* conditions using TLC coupled bioassays (Poonguzhali et al., 2007). *B. vietnamiensis* TVV75ᵀ was isolated from acid sulfate rice soil in South Vietnam, exhibits high nitrogen fixation potential *in vitro,* and produces a novel siderophore compound (Van Tran, 1994; Gillis et al., 1995; Van Tran et al., 1996). In the present study, we confirm that *B. vietnamiensis* TVV75ᵀ and related strains synthesize a diverse suite of AHL molecules, with C_10_-HSL being the dominant molecule, and possess the distinct BviI/R QS circuit in addition to the canonical CepI/R circuit. Specifically, we demonstrate that in *B. vietnamiensis* CBMB40, the *bviI* synthase governs the production of long-chain AHLs, C_10_-, and C_12_-HSLs, while the CepI synthase produces C_6_- and C_8_-HSLs. *B. vietnamiensis* CBMB40 effectively colonizes the tomato rhizosphere, produces AHLs *in situ*, and modulates microbial and plant-associated signaling dynamics, including interspecies communication within the rhizosphere microbiome. Additionally, we provide evidence that QS in CBMB40, potentially through regulation of antibiotic biosynthesis, plays a critical role in its antagonistic activity against plant pathogens.

## 2 Materials and methods

### 2.1 Bacteria and culture conditions

*Burkholderia vietnamiensis* strains were routinely cultured in either Luria Bertani (LB) or King’s B (KB) medium (King et al., 1954) unless otherwise stated. *Escherichia coli* strains, *Chromobacterium violaceum* CV026, and *Erwinia caratovora* subsp. *caratovora* were cultured in LB medium, while *Pseudomonas putida* F117 (pRK-C12) was grown in LB medium containing 4 g NaCl (LBm). *Agrobacterium tumefaciens* NT1 (*traR, tra:lacZ749*) was grown on AB minimal media with 0.5% mannitol (ABM) at 30°C. All bacterial strains were incubated at 30°C, except for *E. coli,* which was grown at 37°C. When required, antibiotics were added to the media at the following final concentrations, in μg per ml: nalidixic acid, 10 for *B. vietnamiensis* CBMB40; Kanamycin, 50 and/or 100 for *P. putida* F117 and *E. coli,* respectively; Ampicillin, 50, and Gentamycin, 20 for *E. coli;* Gentamycin, 20 for *A. tumefaciens* NT1. The fungal cultures used for the antagonism assay were cultured in Potato Dextrose medium at 30°C. A complete list of bacterial strains, AHL indicator strains, and the plasmids used in this study is provided in Table 1.

**Table 1:** List of bacterial strains and plasmids used in this study.

Strain/plasmid	Relevant characteristics	Reference
Indicator strains		
*Agrobacterium tumefaciens* NT1	Ti plasmidless host	Piper et al. (1993)
*Chromobacterium violaceum* CV026	A double mini Tn5 mutant of *C. violaceum* ATCC31532 was used for detecting *N*-acyl-homoserine lactone	McClean et al. (1997)
*Pseudomonas putida* F117	AHL-negative mutant of the wild-type *P. putida* IsoF, a rhizosphere isolate from tomato	Steidle et al. (2002)
*Escherichia coli* MT102	*araD139* (*ara-leu*)*7697* Δ*lac thi hsdR*	Steidle et al. (2002)
*E. coli* strains		
*E. coli* S17-1	Pro^−^Res^−^Mod^+^*recA*	Simon et al. (1983)
*E. coli* HB101	F^−^ hsdR hsdM leu pro thi lacY galK ara xyl rpsL supE recA13	Windle (1986)
*Burkholderia vietnamiensis* strains		
CBMB40	Plant growth-promoting N_2_-fixing strain, wild type, na^r^	Poonguzhali et al. (2007)
SXo-702	N_2_-fixing isolate from the rhizosphere of sorghum	Estrada-de los Santos et al. (2001)
TVV75^T^	N_2_-fixing isolate from the rhizosphere soil of rice	Gillis et al. (1995)
CBMB40-*gfp*1	The gfp derivative of *B. vietnamiensis* CBMB40, GFP^+^, na^r^, Km^r^	This study
ΔCBMB40	AHL-negative mutant of *B. vietnamiensis* CBMB40, Km^r^	This study
Plasmids		
pJM749	*lacZ* reporter fused to a *tra* gene, whose expression is dependent on TraR	Piper et al. (1993)
pRK-C12	pBBR1MCS-5 a broad host range plasmid, carrying P*lasB-gfp* (ASV)-P*lac-lasR*; Gm^r^	Steidle et al. (2002)
pJBA132	pME6031 carrying *luxR*-P*luxR*-P*lux1-gfp* (ASV)-T0-T1; Tet^r^	Steidle et al. (2002)
pRK2013	Mobilizing plasmid, colE1, tra1(RK2), Kan^r^ (Tn903)	Figurski and Helinski (1979)
pUT mini-Tn5	Km^r^ delivery plasmid for Tn5	Herrero et al. (1990)
pFAJ1820	pUT mini Tn5*gusA-pgfp*, Km^r^	Xi et al. (1999)
pJFF350	Omegon-Km transposon containing the aphA kanamycin resistance gene and an *E. coli* origin of replication.	Nemergut and Schmidt (2002)

### 2.2 AHL extraction and GC–MS analysis

AHLs extraction and purification from *B. vietnamiensis* were performed following previously described procedures (Cha et al., 1998; Burton et al., 2005). Essentially, spent supernatants from cultures of *B. vietnamiensis* grown in 1 L LB broth to an OD_600_ of 1.3 were extracted with dichloromethane, and the residue was resuspended in 100 μL methanol and stored at −20°C before GC–MS analysis. GC–MS analyses were performed using a model CP-3800 GC system (Varian, Inc., Mitchell Drive, Walnut Creek, United States) interfaced to a 1,200 L Quadrupole MS–MS detector (Varian) fitted with a BPX5 fused-silica capillary column (30 m x 250 μm i.d. and 0.25 μm film thickness, Varian Inc., United States). Conditions were as follows: injector temperature: 200°C; transfer line temperature: 280°C; electron energy: 70 eV; injection volume: 1 μL. The GC was programmed as follows: 5 min at 150°C and increasing at 15°C min^−1^ to 275°C and operated in a split mode. The carrier gas was He at 0.8 mL min^−1^. The mass spectrometer was run in full scan mode (*m/z* 15–800) and in SIM mode (*m/z* 143). The total ion chromatogram (TIC) spectra containing an ion at *m/z* 143 were compared with the product mass spectra of the corresponding synthetic AHL standard. Synthetic HSLs, namely *N*-hexanoyl HSL (C_6_-HSL), *N*-heptanoyl HSL (C_7_-HSL), *N*-octanoyl HSL (C_8_-HSL), *N*-decanoyl HSL (C_10_-HSL), *N*-dodecanoyl HSL (C_12_-HSL), and 3-oxo-hexanoyl HSL (3-oxo-C_6_-HSL), used as reference standards were obtained from Fluka and Sigma (Sigma Aldrich Co., St. Louis, MO, United States). Data analysis was carried out with MS workstation SP1 version 6.5 (Varian).

### 2.3 DNA manipulations and analysis

The presence of two QS systems, *cepI/R* and *bviI/R,* and hence the genes in *B. vietnamiensis* strains, was determined by polymerase chain reaction (PCR) with specific primers (Gotschlich et al., 2001; Lutter et al., 2001). Details of the primers used are mentioned in Supplementary Table S1. The PCR products were sequenced using the same primers directly and analyzed. PCR products were purified using a QIAquick PCR purification kit, and DNA sequencing was carried out by Sol Gent Co., Ltd. (Daejeon, Republic of Korea) in an automatic DNA sequencer (ABI Prism 3720xl DNA analyzer, Tokyo, Japan) using Bigdye^™^ terminator method. The products were purified using the Montage dye removal kit (Millipore).

*In silico* analysis of nucleotide and protein sequences was performed using BLASTx and BLASTp searches available online at NCBI. DNA sequences were analyzed and aligned using the DNASTAR –SEQMAN program, and the overlapping protein sequences were aligned, and the consensus sequence was computed using Clustal V software (Higgins et al., 1992). A Maximum likelihood phylogenetic tree from the aligned sequences was constructed using 1,000 bootstrap replication values. Evolutionary trees for the datasets were inferred by the Neighbor-Joining method by Saitou and Nei (1987) using the Neighbor-Joining program, MEGA version 3.10 (Kumar et al., 1993). The *bviI* and *bviR* gene sequences from the *B. vietnamiensis* strains (CBMB40, SXo-702, and TVV75^T^) analyzed in this study have been deposited in the GenBank/NCBI database under accession numbers EF553320 to EF553325. Similarly, the *cepI* and *cepR* gene sequences have been submitted under accession numbers EU033994, EU033997, EU033998, EU033999, EU034002, and EU034003.

Plasmid DNA was isolated with the QIAGEN miniprep kit (QIAGEN Korea Ltd., Seoul). Preparation of chromosomal DNA, restriction enzyme digestion, agarose gel electrophoresis, ligation, and transformation to *E. coli* were performed using standard procedures (Sambrook et al., 1989) unless specified.

### 2.4 Construction of recombinant plasmids of *Burkholderia vietnamiensis* CBMB40

To create expression plasmids of the AHL synthases *bviI* and *cepI* in *E. coli*, the full-length sequences of the respective genes were amplified from *B. vietnamiensis* CBMB40 genomic DNA using PCR primer sequences listed in Supplementary Table S1. The amplified products were purified and cloned into the pCR^™^2.1-TOPO^®^ TA blunt vector (Invitrogen, Korea) as per manufacturer’s instructions followed by transformation into competent *E. coli* DH5α. The recombinant vectors, designated TOPO-TA-bviI, TOPO-TA-cepI, and the vector pQE31 (Qiagen, Korea) were digested with HindIII/BamHI. Subsequently, the purified inserts and the linearized vector were ligated and transformed into *E. coli* DH5α through electroporation. The resulting plasmids were designated as pQE31-BviI and pQE31-CepI, and at each step, the plasmids were verified by sequencing.

*B. vietnamiensis* CBMB40 was tagged with GFP by introducing the plasmid pFAJ1820 (Xi et al., 1999) through triparental matings (Unge et al., 1998). Fluorescent ex-conjugant CBMB40-*gfp1* that constitutively expressed *gfp* checked through PCR, had higher relative fluorescence activity and grew indistinguishably from the parental strain in LB and KB medium, was selected for inoculation experiments. The *gfp* derivatives were also checked for AHL production using cross-streak assays against CV026 (Supplementary Figure S1).

An AHL-lacking mutant of *B. vietnamiensis* CBMB40 (ΔCBMB40) was obtained using random transposon mutagenesis via conjugation with *E. coli* S17-1 (pJFF350) that carried an Omegon-Km transposon (Nemergut and Schmidt, 2002) and *E. coli* HB101 (pRK2013) as the helper strain. In brief, a bank of random insertion mutants was made by mixing the cells grown in appropriate medium with antibiotics at a ratio of 1:5:1 for donor, recipient, and helper strain, washed and suspended in phosphate-buffered saline (PBS, pH 7.4). The mating mixtures were spotted directly onto LB medium without any antibiotics onto filter paper discs placed on it and grown at 30°C overnight. The mutant bank obtained on selective minimal media with azelaic acid (PCAT) containing nalidixic acid and kanamycin was tested for its ability to produce AHLs using *C. violaceum* CV026 as the reporter. Putative AHL clone lacking the ability to induce violacein production in CV026 was selected (ΔCBMB40) and further characterized.

### 2.5 Bioassays for AHL production

To dissect the AHL production profile in CBMB40, the extraction of AHLs from the culture supernatants and TLC bioassays with *C. violaceum* CV026 and *A. tumefaciens* NT1 (*traR, tra:lacZ749*) were performed as described previously (Poonguzhali et al., 2007). Alternatively, the AHL extracts from CBMB40 were used for the estimation of violacein activity induced in *C. violaceum* CV026 according to Blosser and Gray (2000). Cross-feeding bioassay was conducted using *C. violaceum* CV026 and *P. putida* F117 (pRK-C12) to screen for an AHL deletion mutant (ΔCBMB40) and for visualizing rhizosphere intergeneric communication. For CV026, AHL production was detected by streaking the strain to be tested and the indicator strain in parallel in LB agar plates and inspecting for purple pigment production during incubation at 30°C. The extracts from CBMB40 were spot inoculated onto LBm medium (0.7% agar) seeded with *P. putida* F117 (pRK-C12) or *E. coli* MT102 (pJBA132). After incubating the plates at 30°C for 24 h, the fluorescence halo formed was checked under UV in the dark.

### 2.6 Plant material, growth conditions, and bacterial inoculum

Tomato seeds (*Lycopersicon esculentum* L. cv. Mairoku) were obtained from Sokato Korea (Seoul), and red pepper (*Capsicum annuum* L. cv. Barodda) seeds were obtained from New Seoul Seed Company (Kongju). Seeds surfaces sterilized with 70% ethanol for 3 min and 5% sodium hypochlorite (with 0.6% Tween20) for 20 min, followed by subsequent rinses in sterile distilled water 5 times, were allowed to pre-germinate on moist filter papers (7 days at 24°C in dark). Germinated seedlings were transferred to PhytaTrays (Sigma, United States) filled with 250 g of sterilized sand and 30 mL of sterile nutrient solution (Simons et al., 1996). Six plants per tray and four trays per treatment were maintained. After covering and sealing the Phyta trays with parafilm, the plants were incubated in a growth chamber (Conviron^™^, Republic of Korea) with a circadian cycle of 14 h (25 ± 1°C)/10 h (20 ± 1°C) and 70 ± 5% humidity.

For inoculation studies, *B. vietnamiensis* CBMB40, both wild, and its derivatives were proliferated in King’s B medium (OD_600nm_ = 1.0) supplemented with antibiotics when appropriate, pelleted (8,000 *× g*, 10 min at 4°C), washed, and resuspended in sterile 0.01 M MgSO_4_ (10^8^ CFU/ ml). Seeds treated with 0.01 M MgSO_4_ served as a control. Surface-sterilized seeds or germinated seedlings were immersed in the bacterial suspension for 4 h with shaking at room temperature.

### 2.7 Plant experiments to check colonization, root adherence, AHL-mediated signaling in the rhizosphere

To check colonization of tomato roots by CBMB40, tomato seedlings treated with CBMB40-*gfp1* were transferred to Phytatrays and incubated. Samples from at least three seedlings were observed at intervals after 2 weeks of inoculation. Plants were inoculated with either CBMB40, CBMB40-*gfp1,* or control, and roots were washed in sterile phosphate-buffered saline (PBS). The samples were kept at 4°C overnight before processing and mounted using Vectashield (Vector Laboratories Inc., Burlingame, CA, United States) mounting medium under a coverslip for observation.

While for checking the root adherence by wild type or transformants, treated seedlings (red pepper) grown in growth pouches were harvested after 15 days, and their root length was measured. Subsequently, the root system was washed in sterile distilled water to remove non-adhering bacteria, and roots were blotted dry on filter paper, weighed, and cut into 1-cm sections. Bacterial population adhering to the root sections was removed by vortexing (60 s) and sonication (5 min) in 5 mL PBS and plated onto PCAT media with and without antibiotics as required and incubated.

To visualize AHL-mediated signaling between *B. vietnamiensis* CBMB40 and *P. putida* F117 in the tomato rhizosphere, treated seeds were transferred to Phytatrays and grown in a growth chamber. The plants were inoculated with 3 mL of individual bacterial strain grown to logarithmic phase or a mixture of two strains at a 1:1 ratio, near the root zone, 10 days after sowing, i.e., 3 to 4 days after germination (Steidle et al., 2002). Root samples were washed in sterile PBS and observed immediately under a coverslip with the mounting medium at 5 and 11 days after inoculation.

### 2.8 Confocal laser scanning microscopy

Microscopic examinations and image acquisitions were performed using a Leica TCS SP2 confocal system (Leica Microsystems Heidelberg GmbH, Manheim, Germany) equipped with an Ar ion laser (Gfp: excitation, 488 nm; emission filter BP 500–530). Image acquisitions were carried out using a × 40 objective (N.A ∼ 0.75) or ×63 oil immersion objective (N.A ∼ 1.40) and processed using the standard software package with the CLSM system (Version 2.5.1227a).

### 2.9 Exoenzyme assays, siderophore production, swarming motility, and antagonism against *Erwinia caratovora* and fungal pathogens

Protease activity was tested on tryptone soy agar plates with skim milk prepared according to Harris et al. (1998). Cultures (logarithmic phase) grown in LB medium were spot inoculated onto the plates and incubated at 30°C for 48 h to inspect the clearance zones produced by the enzyme activity. Swarming assays were performed on nutrient broth with 0.5% glucose and 0.5% agar. One microlitre of overnight culture grown in liquid medium was point inoculated into the center of the agar plates and incubated for 18–42 h (Lewenza et al., 2002).

For checking the antagonism against *E. caratovora* subsp. *caratovora,* 10 μL of the test strain was spot inoculated at the center of the plates seeded with an overnight culture of the pathogen and incubated at 30°C for 24–48 h to observe the inhibition zones. *In vitro* antagonism against fungal pathogens was tested in a dual culture plate assay. Actively growing fungal mycelial plug allowed to grow on PDA for 48 h at 28 ± 2°C was challenge inoculated with the test strain onto the four corners of the media (spot inoculated or streak) and incubated for 7 d, after which the radial diameter of the fungal growth was measured. Triplicate plates were maintained per fungal pathogen, and the plated inocula with fungal pathogen alone served as control.

### 2.10 *In vitro* maceration of potato tubers

Potatoes purchased from the local market were washed thoroughly with running tap water and sterilized with sodium hypochlorite (0.5%) for 10 min, extensively rinsed with sterile distilled water, and finally dried under sterile conditions. Strains were cultivated overnight, and the cell pellets (10,000 *xg*/10 min/4°C) were suspended and diluted in 0.85% NaCl solution to an OD_600nm_ of 0.8. Whole tubers were inoculated with the pathogen or equal volumes of test strain and the pathogen (200 μL), which is injected using a micropipette tip (Uroz et al., 2003) before incubation at 25°C under a humid atmosphere (over 90% humidity) for 3 days. The results of the inoculation were assessed by visual inspection after slicing the tubers, and the diameter of the lesions produced was numerically recorded.

### 2.11 Plant experiments to check the pathogenicity of *Phytopthora capsici*

Surface-sterilized red pepper seeds treated with bacterial suspension were grown in multi-well trays filled with sterilized potting substrate (Wonjo-Mix bed soil, Nong-Kyung Co., Ltd., Jincheon-gun, Chungbuk, Republic of Korea) before transplanting them to non-sterile potting substrate in pots and growing under controlled conditions. The moisture content was maintained at 80% water holding capacity throughout the experiment period, and nutrient solution (5 mL) was added to each pot at weekly intervals. The pots were arranged in a completely randomized design with six plants per treatment and two replications for each treatment. Bacterial inocula were applied near the root zone at the time of planting (2 mL) and 10 days after transplanting (10 mL). A zoospore suspension of *Phytophthora capsici* KACC 40157, cultured in PDA at 10^5^ zoospores/ml, was used to inoculate the plants at the eight-leaf stage using the stem wound technique by Hwang et al. (1996). The stems of pepper plants were wounded by making a 1-cm longitudinal slit approximately 2 cm above the soil surface. Sterile cotton plugs soaked in zoospore suspension were placed on the wounded sites and secured with plastic tape. Plants were observed daily for symptom development. The experiment was repeated twice, and the data presented are from one representative experiment.

### 2.12 Statistical analysis

All experiments were conducted at least twice, with three replicate samples per test condition. The results presented are from a representative experiment. Data are expressed as the mean ± standard error of the mean (SEM) for each group. One-way analysis of variance (ANOVA) was used to determine statistically significant differences among treatment groups. A *p*-value of less than 0.05 was considered statistically significant. Where applicable, individual treatments and replicate comparisons were evaluated using the Student’s *t*-test.

## 3 Results

### 3.1 Plant-associated *Burkholderia vietnamiensis* produces a mixture of AHLs and possesses two QS bviI/R and cepI/R circuits

**Figure 1:**
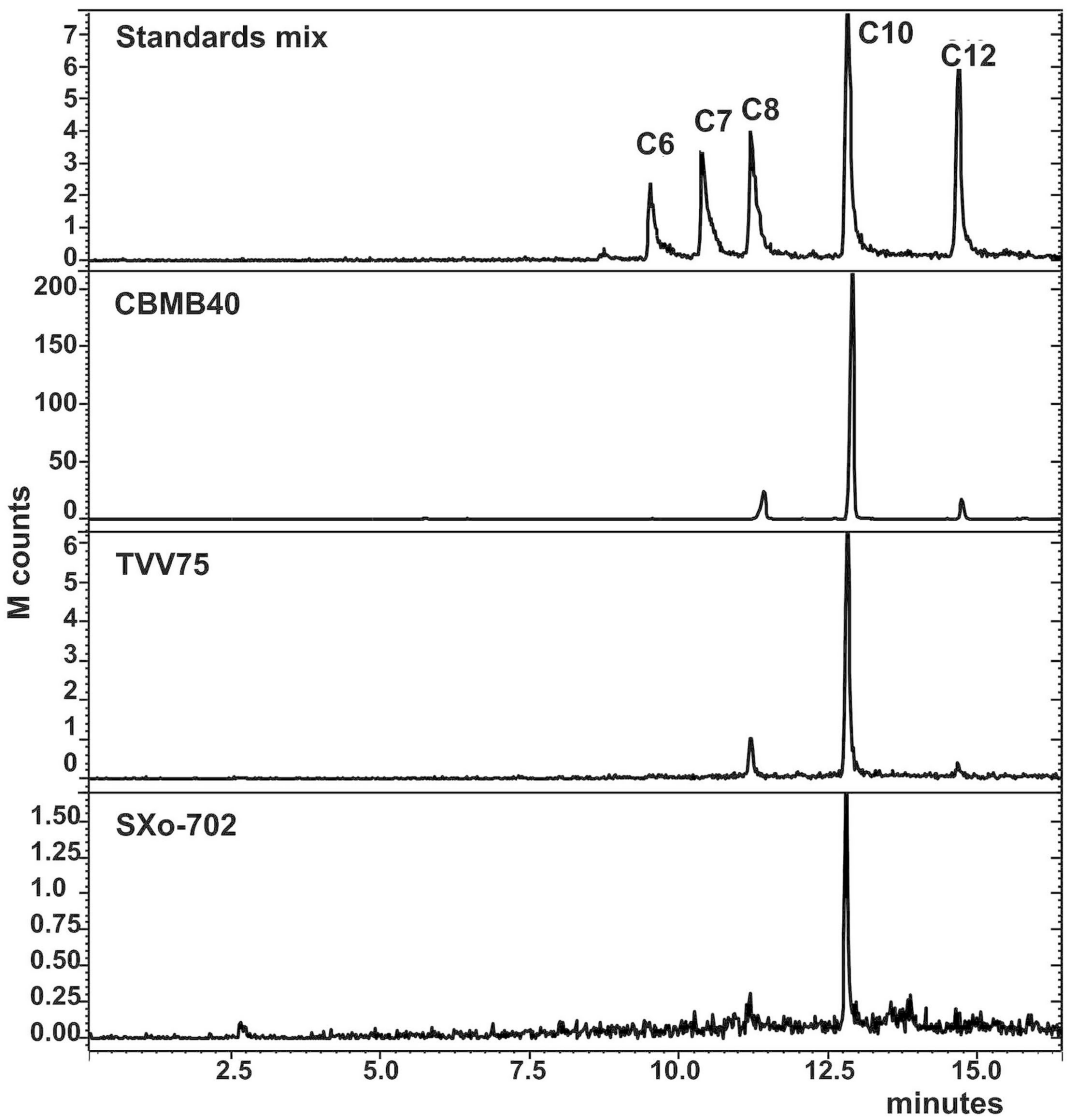
GC–MS chromatograms of the extracts of cell-free supernatant of *B. vietnamiensis* strains obtained at a selected ion monitoring of *m/z* 143. The chromatograms of the standard mix (100 ppm) are shown to compare the retention times. The extracts of culture supernatants were dissolved in methanol and examined. C_6_—C_6_-HSL, C_7_—C_7_-HSL, C_8_—C_8_-HSL, C_10_—C_10_-HSL, C_12_—C_12_-HSL. All three strains of *Burkholderia* examined showed characteristic Chromatogram similar to C_8_-HSL, C_10_-HSL, and C_12_-HSL, with C_10_-HSL remaining prominent.

TLC analysis of solvent-extracted culture supernatants from *B. vietnamiensis* strains TVV75^T^, SXo-702, and CBMB40, combined with biosensor-based detection using *C. violaceum* CV026 and *A. tumefaciens* NT1, confirmed the production of multiple AHLs in all three strains (Poonguzhali et al., 2007). To unambiguously identify the specific AHLs produced by these strains, GC–MS analysis was employed due to its superior separation capability, sensitivity, and structural elucidation power. AHLs were identified based on mass spectral fragmentation patterns, retention indices, and comparison with synthetic standards. The mass spectra of the AHLs identified showed a characteristic fragmentation pattern with a base peak at *m/z* 143, along with other characteristic ions at *m/z* 102, 71, 57, 43, and a small molecular ion peak [M^+^] consistent with previous reports (Cataldi et al., 2004; Wagner-Döbler et al., 2005). A profile of three AHL molecules, C_8_-, C_10_-, and dodecanoyl (C_12_-) HSLs, could be identified in all the strains analyzed, with C_10_-HSL being the most abundant (Figure 1). Although strong TLC signals suggested the presence of C_6_-HSL, this molecule was only detected in trace amounts or not at all by GC–MS, suggesting that bioassays may detect compounds below the GC–MS detection threshold or structurally similar AHL analogs.

**Figure 2:**
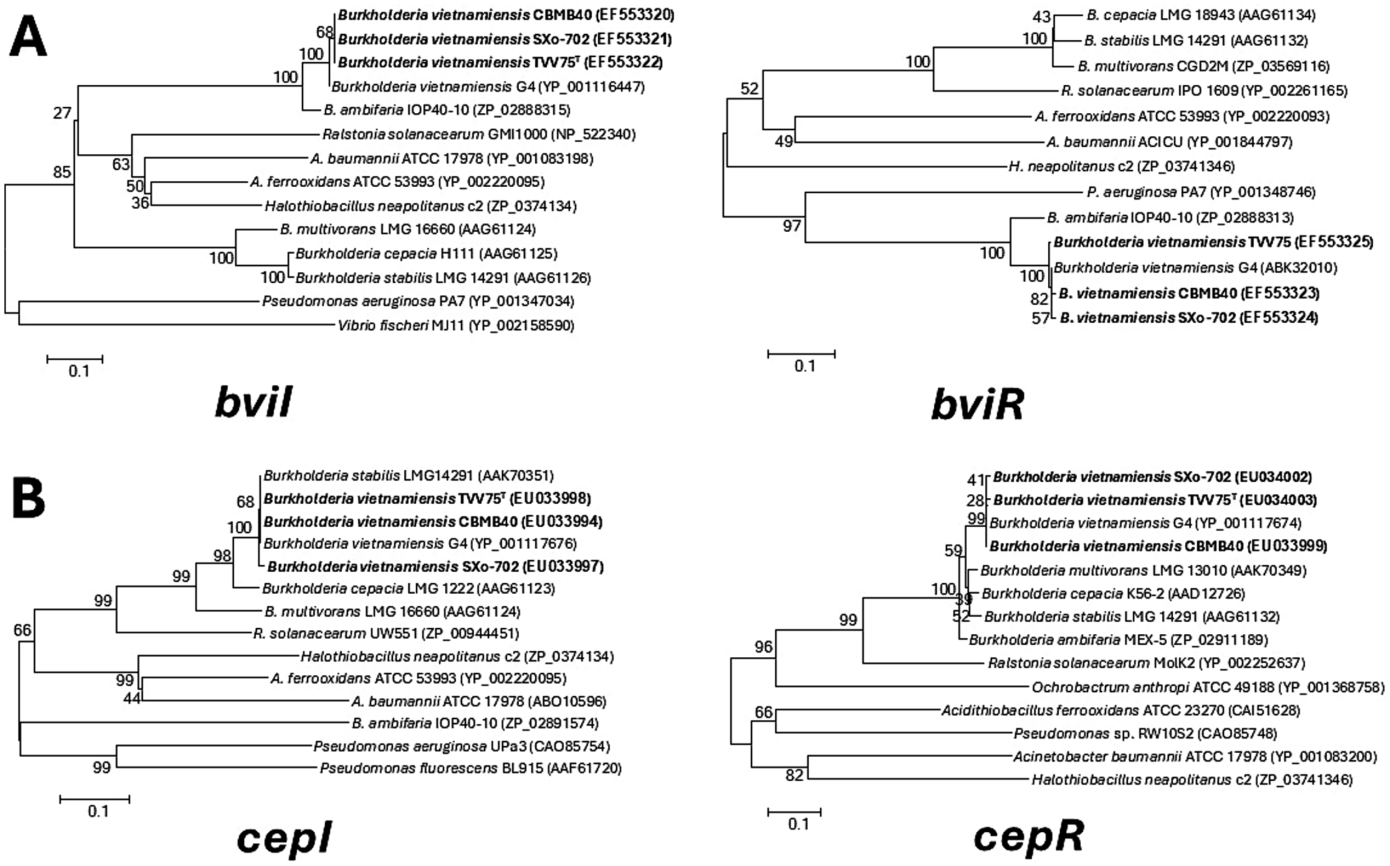
Phylogenetic analysis of *Burkholderia vietnamiensis* strains based on quorum sensing synthase (I) and receptor (R) proteins. Trees were constructed using the neighbour joining algorithm based on the amino acid sequences of **(A)** BviI and BviR and of **(B)** CepI and CepR. Bootstrap analysis was performed with 1,000 replicates, and values are indicated at the nodes and the scale bar indicates 0.1 substitutions per amino acid position.

In addition to the well-characterized *cepIR* QS system, *B. vietnamiensis* harbors a second distinct circuit *bviIR* that appears to govern the production of the majority of AHLs produced (Conway and Greenberg, 2002; Malott and Sokol, 2007). Using gene-specific primers, PCR amplification, and sequencing of the *cepI/R* and *bviI/R* genes in these strains revealed near-complete sequence conservation with previously characterized homologs. The deduced amino acid sequences of *bviI* from *B. vietnamiensis* showed a high percentage of similarity (97.5–99.4%) within them, and they showed close similarity to the *B. vietnamiensis* G4 AHL synthase proteins (95.8–97.0%). These genes also showed 86.3–88.3% (*bviI*) and 86.6–89% (*bviR*) sequence identity to the *bamI/R* system of *Burkholderia ambifaria* (Figure 2). Similarly, the cepI/R proteins of the strains were identical with *B. vietnamiensis* G4, showing 99.3–100% sequence similarity. The *CepI* showed 98.8–99.4% similarity with *CepI* from *Burkholderia stabilis,* while the CepR protein had 90.5–94.3% similarity to *CepR* from *B. cepacia* (Figure 2).

Together, these results demonstrate that plant-associated *B. vietnamiensis* strains possess two conserved QS circuits (*cepI/R* and *bviI/R*) and produce a consistent mixture of AHLs, with C_10_-HSL as the predominant signal molecule. The high degree of conservation in both AHL profiles and QS gene sequences underscores the evolutionary stability of these regulatory systems in rhizosphere-adapted strains.

### 3.2 AHL QS system in plant growth-promoting *Burkholderia vietnamiensis* CBMB40

**Figure 3:**
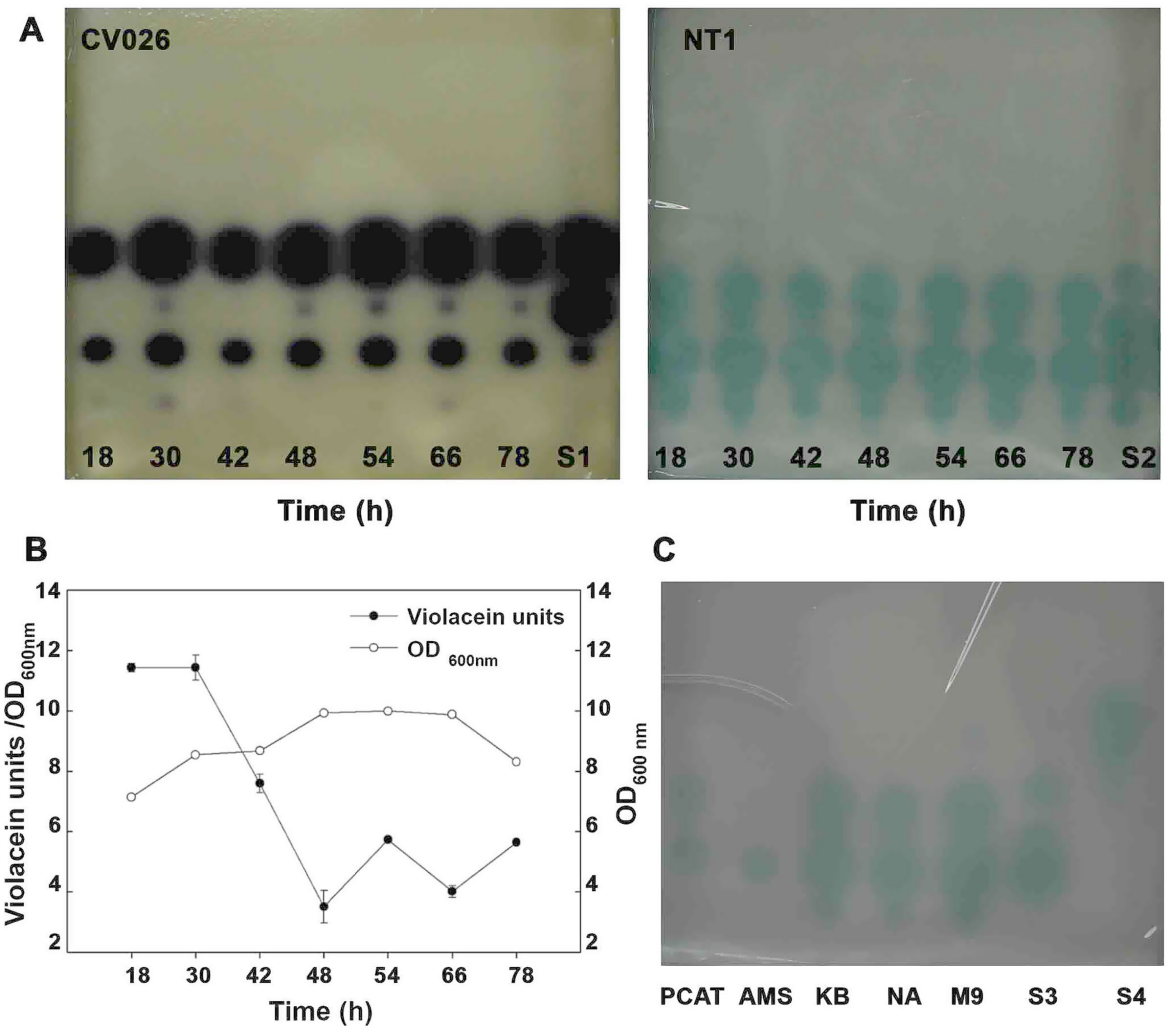
Quorum-sensing signal production in *B. vietnamiensis* CBMB40 as a function of growth and different carbon sources. **(A)** Culture supernatants were extracted from CBMB40 grown in LB at the indicated time points and separated via TLC coupled bioassays with *C. violaceum* CV026 and *A. tumefaciens* NT1 (*traR, tra:lacZ749*). **(B)** Violacein production in the culture supernatant extracts at different time intervals was measured using *C. violaceum* CV026. **(C)** TLC coupled bioassay with *A. tumefaciens* NT1 (*traR, tra:lacZ749*) for the culture supernatant extracts from different media with different carbon sources. PCAT: PCAT media with azelaic acid, AMS: AMS with Succinate, KB: Kings’ B media, NA: NA with 1% methanol, M9: M9 minimal media with glucose. S1–S4 are mixtures of standards. S1, S3- a mixture of C_6_-, C_7_-, and C_8_-HSLs, S2- mixture of C_6_, C_7_, C_8_, and C_10_-HSLs, S4–3-oxo-C_6_-HSL.

To investigate the dynamics of AHL production in *B. vietnamiensis* CBMB40 and its relationship to bacterial growth, TLC was performed using the biosensors *C. violaceum* CV026 and *A. tumefaciens* NT1 (*traR*, *tra:lacZ749*). AHL activity was visualized by comparing the diameters and migration patterns of detected spots against a dilution series of AHL standards. AHLs were consistently detected only during the mid-logarithmic to late exponential growth phases (Figure 3A), with no substantial variation in AHL concentration across different time points during these phases. In contrast, when violacein production in CV026 was used as a functional readout of AHL activity in culture supernatants collected at various growth phases, a growth phase-dependent trend emerged. Specifically, violacein production normalized to cell density (OD_600nm_) decreased notably after the late logarithmic phase (Figure 3B), suggesting that AHL biosynthesis in CBMB40 is not constitutive. These results indicate that AHL production is regulated in a growth phase-dependent manner, a pattern previously reported in other bacteria, such as *Yersinia enterocolitica* (Throup et al., 1995) and various *Enterobacteriaceae* isolates (Ravn et al., 2001). Additionally, TLC bioassays revealed that the number and type of AHLs produced varied with growth stage. Notably, a compound co-migrating with the C_7_-HSL standard appeared only during later growth phases, further supporting growth phase-dependent modulation of AHL synthesis.

To determine whether carbon source availability influences AHL production in CBMB40, the strain was cultured in different media: PCAT, AMS, or minimal media with glucose (M9), as well as in complex media such as nutrient agar (NA) with 1% methanol and KB medium. AHL profiles were analyzed by both TLC and GC–MS. Significant variation in AHL composition was observed across media conditions (Figure 3C). Surprisingly, the presence of a molecule migrating with C_10_-HSL was limited to complex media or minimal media containing glucose. In contrast, cultures grown on succinate produced only a single AHL migrating with C_6_-HSL. GC–MS analysis corroborated the TLC findings, confirming distinct AHL profiles in response to different carbon sources (Supplementary Figure S2). Intriguingly, growth on azelaic acid yielded a compound with mass spectral features characteristic of AHLs; however, this compound could not be conclusively identified due to a lack of matching standards. This may indicate either the production of a novel AHL or limitations in detection sensitivity.

Taken together, these results demonstrate that both growth phase and carbon source significantly influence AHL production in *B. vietnamiensis* CBMB40, affecting both the diversity and relative abundance of the AHL molecules synthesized.

### 3.3 *Burkholderia vietnamiensis* CBMB40 *bviI* directs the synthesis of major AHLs in *Escherichia coli*

**Figure 4:**
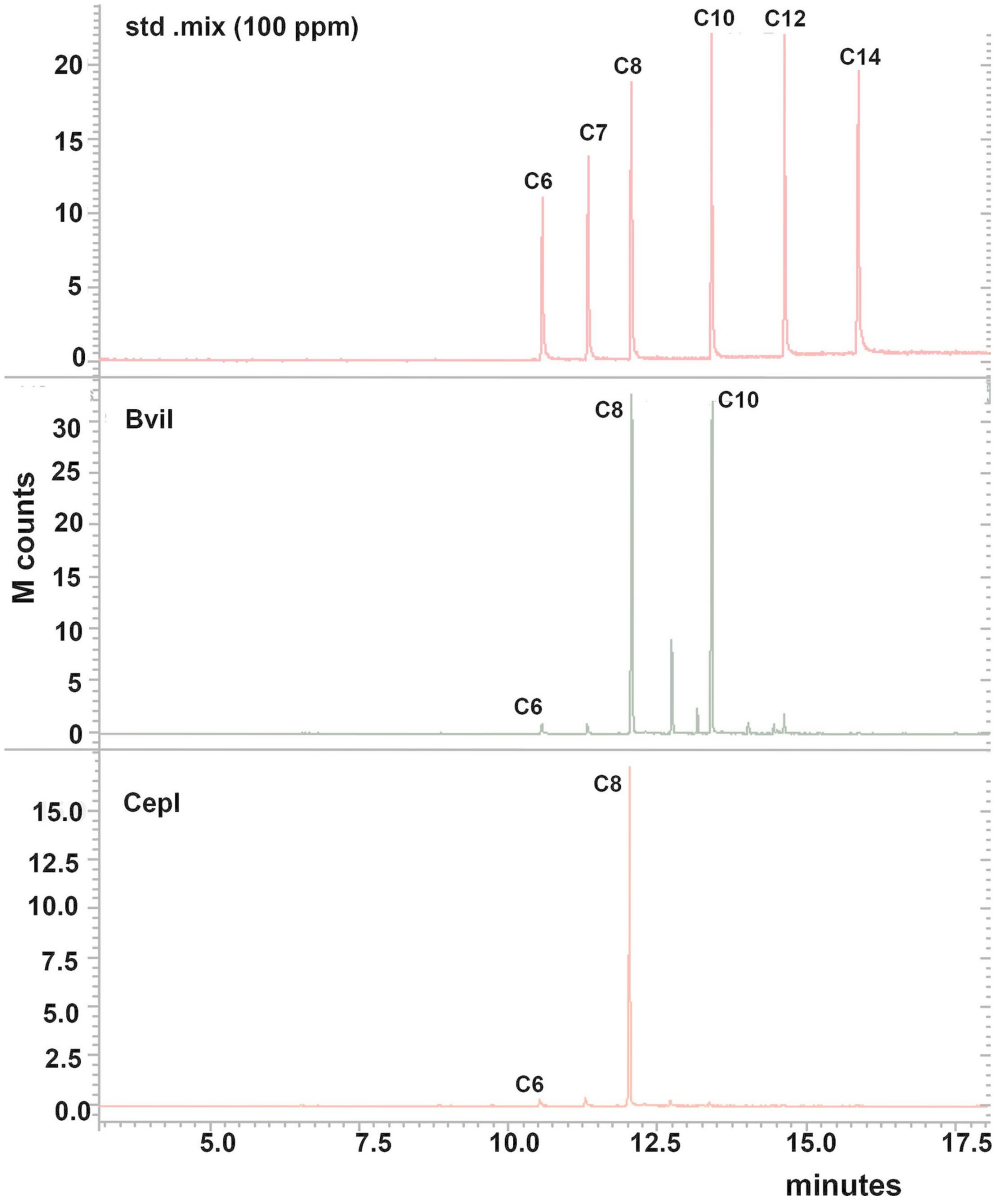
GC–MS chromatograms of the extracts of cell-free supernatant of *E. coli* DH5α bviI-1 and cepI-5 strains obtained at a selected ion monitoring of *m/z* 143. The chromatograms of the standard mix (100 ppm) are shown to compare the retention times. The extracts of culture supernatants were dissolved in methanol and examined. C_6_—C_6_-HSL, C_7_—C_7_-HSL, C_8_—C_8_-HSL, C_10_—C_10_-HSL, C_12_—C_12_-HSL, C_14_—C_14_-HSL. The strain with BviI-1 produced most AHLs produced by the parent strain *B. vietnamiensis* CBMB40, while the CepI-4 strain showed only C_6_- and C_8_-HSLs.

*Burkholderia vietnamiensis* CBMB40 encodes two AHL-dependent QS circuits, *bviI* and *cepI*, which contribute to the synthesis of a range of AHLs. To determine the specific AHLs produced by each synthase, the *bviI* and *cepI* genes from CBMB40 were PCR-amplified, cloned into the expression vector pQE31, and designated as pQE31-*bviI* and pQE31-*cepI*, respectively. Plasmid constructs were verified by DNA sequencing and subsequently transformed into *E. coli* DH5α for heterologous expression. Expression of AHLs in the recombinant *E. coli* strains was evaluated using the biosensor CV026. Both *E. coli* DH5α:*bviI*-1 and DH5α:*cepI*-5 induced violacein production in CV026, confirming AHL activity. In contrast, the control strain harboring the empty vector (pQE31) showed no detectable AHL activity. To identify the specific AHLs synthesized, cell-free supernatants from log-phase cultures of *E. coli* DH5α:*bviI*-1 and *E. coli* DH5α:*cepI*-5 were extracted with dichloromethane and subjected to GC–MS analysis. The DH5α:*bviI*-1 strain produced C_8_-HSL, C_10_-HSL, and C_12_-HSL, whereas DH5α:*cepI*-5 produced only C_8_-HSL (Figure 4). No AHLs were detected in the control strain, confirming that AHL production was dependent on expression of the respective QS genes. These results demonstrate that *bviI* is the primary AHL synthase in *B. vietnamiensis* CBMB40, responsible for the production of multiple AHL species, while *cepI* appears to contribute to a narrower spectrum, predominantly short-chain AHLs, such as C_6_- and C_8_-HSLs.

### 3.4 *Burkholderia vietnamiensis* CBMB40 colonizes, produces AHLs *in situ,* and modulates signaling in the rhizosphere

**Figure 5:**
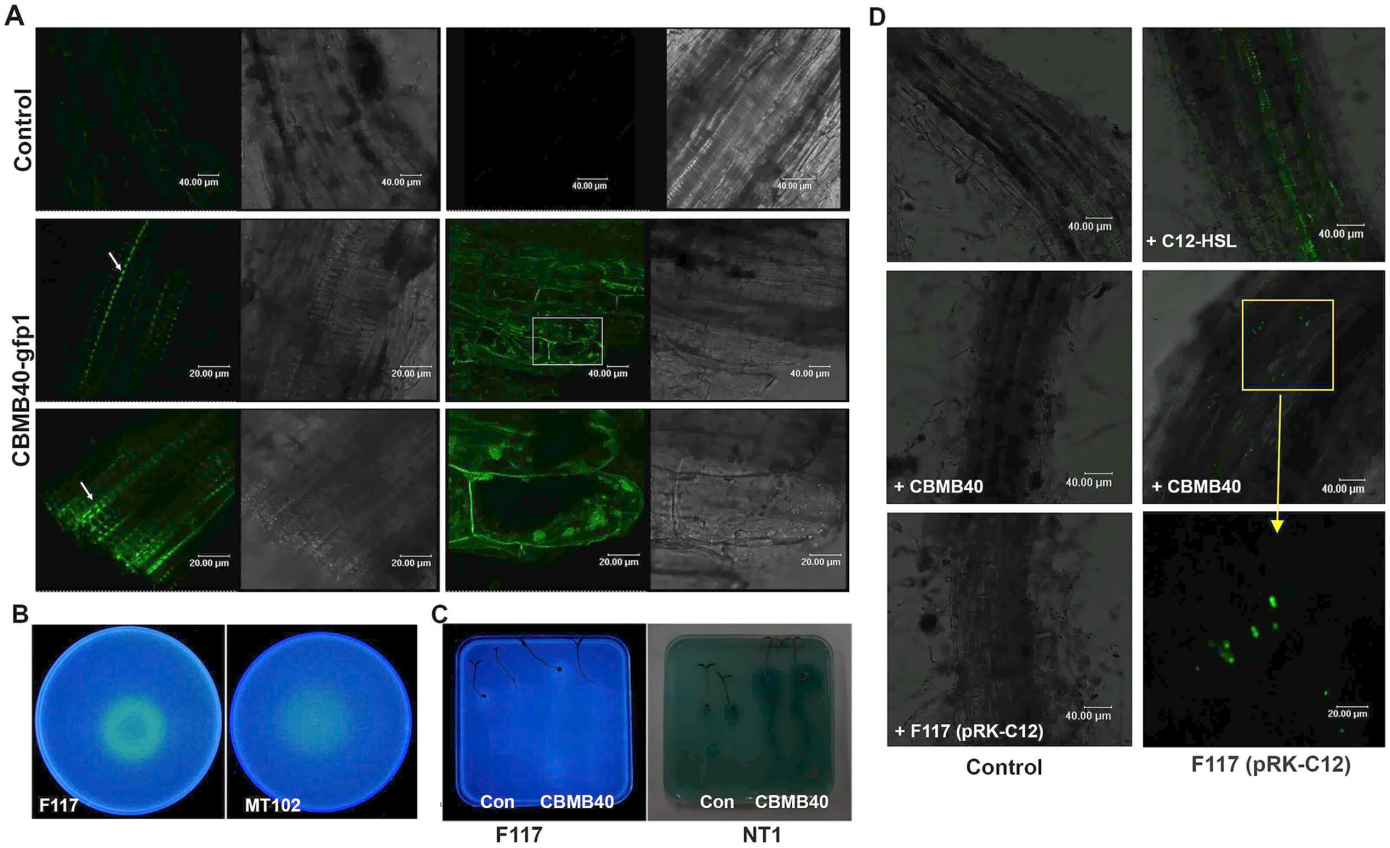
*B. vietnamiensis* CBMB40 colonizes the rhizosphere of tomato plants and produces AHLs *in situ* and communicates via AHLs in the rhizosphere **(A)**. Colonization of tomato root surface (left) and intercellular spaces (right) by *B. vietnamiensis* CBMB40-*gfp*1. Phase contrast and fluorescence micrographs of CLSM showing a row of single cells on the root surface and the point of emergence of lateral roots (white arrows), while no cells are present in the control roots. Cells can also be seen in the intercellular layers (right), and the last right panel is the enlarged view of the region (white rectangle) shown. **(B)** AHLs produced by CBMB40 detected using the indicator strains *P. putida* F117 (pRK-C12) and *E. coli* MT102 (pJBA132) under *in vitro* conditions. The extracts from CBMB40 were inoculated into plates containing *P. putida* F117 (pRK-C12) (left) and *E. coli* MT102 (pJBA132) (right). **(C)** AHLs produced by CBMB40 *in planta*. Seedlings of tomato from treated and untreated seeds were transferred to indicator plates prepared with *P. putida* F117 (pRK-C12) (left) and *A. tumefaciens* NT1 (right). Two seedlings on the left are from uninoculated seeds, and the other two are from treated ones. Both the indicators detected some quorum-sensing mimic compounds from tomato as indicated by the positive response. **(D)** Intergeneric communication between *Burkholderia* and *Pseudomonas* in the rhizosphere of tomato plants. The panels indicate CLSM images of tomato roots without any bacterial inoculation (top left) and roots inoculated with CBMB40, *P. putida* F117, C_12_-HSL, *P. putida* F117, and CBMB40 + F117. An enlarged view of the area pointed to shows the presence of fluorescing cells in the rhizosphere.

The plant growth-promoting *B. vietnamiensis* CBMB40 demonstrated effective colonization of the tomato rhizosphere. Visualization of roots inoculated with GFP-tagged CBMB40 (CBMB40-*gfp1*) by confocal microscopy revealed bacterial cells distributed along the root surface, particularly forming linear strings within the depressions of the root epidermis (Figure 5B). Colonization was most prominent at cell junctions between rhizodermal cells and at lateral root emergence zones. While root hairs were well developed at this stage, no bacterial cells were observed adhering to them. Surface-sterilized root sections showed fluorescence in the intercellular spaces of the epidermis (Figure 5B), confirming the presence of CBMB40 cells. Such fluorescence was absent in control plants (Figure 5A). Although no colonization of internal cortical cells or xylem tissue was observed, the localization at epidermal intercellular spaces may indicate early stages of endophytic colonization.

Previous work confirmed that CBMB40 produces AHLs, which were detectable *in planta* using TLC assays and AHL biosensor-based detection in canola seedlings (Poonguzhali et al., 2007). In the current study, further assays were performed to confirm AHL production *in situ* and investigate whether CBMB40 alters plant-derived signal mimics. AHL detection assays using the biosensor strains *Pseudomonas putida* F117 (pRK-C12) and *E. coli* MT102 (pJBA132), both sensitive to long-chain AHLs, showed that CBMB40 produced AHLs at detectable levels, inducing GFP expression in these strains under UV illumination (Figure 5C). This assay was conducted as a prerequisite to check the AHLs by *Burkholderia* in rhizosphere colonized conditions. Moreover, tomato plants grown under gnotobiotic conditions, both control and inoculated with CBMB40, induced GFP and *β*-galactosidase expression in *P. putida* F117 (pRK-C12) and *Agrobacterium tumefaciens* NT1 (tra:lacZ749), respectively (Figure 5C), indicating active signal exchange between the plants and indicator strains.

Interestingly, analysis of methanolic extracts from uninoculated tomato roots revealed the presence of an AHL-like compound; however, its retention time and mass spectrum did not correspond to any known AHL standards used in this study. Notably, this compound was absent or possibly below the detection limit in extracts from *B. vietnamiensis* CBMB40-inoculated plants. In contrast, extracts from inoculated plants exhibited a distinct spectral profile, with retention times and fragmentation patterns suggestive of long-chain AHLs, including C_8_-, C_10_-, and C_12_-HSLs (Supplementary Figure S3). Despite these observations, definitive identification and quantification of individual AHLs in plant extracts were hindered by spectral overlap and potential interference from background plant-derived metabolites. These matrix effects likely obscured the signals of bacterial AHLs, especially those present in low concentrations. While both biosensor assays and GC–MS analysis were inconclusive in definitively identifying AHLs within plant tissues, the overall experimental aim of determining whether CBMB40 produces AHLs under rhizosphere conditions and whether these molecules are perceived by the plant was effectively supported by the combined data. To strengthen these findings, future studies should incorporate additional purification steps to reduce background plant metabolites and enrich AHL fractions before analysis. Integrating transcriptomic or reporter-based approaches to assess plant responses to AHL exposure would also provide complementary evidence for signal perception and downstream functional outcomes.

To investigate potential intergeneric communication via AHLs in the rhizosphere, tomato roots were co-inoculated with CBMB40 and the AHL biosensor strain *P. putida* F117 (pRK-C12). Confocal microscopy revealed strong GFP fluorescence along the rhizoplane only in co-inoculated plants. No fluorescence was observed when either strain was inoculated individually, indicating that GFP induction was specifically triggered by CBMB40-derived AHLs and not by plant signals (Figure 5D). Collectively, these results demonstrate that *B. vietnamiensis* CBMB40 can successfully colonize the tomato rhizosphere, produce long-chain AHLs *in situ*, and modulate microbial and plant-associated signaling dynamics, including interspecies communication within the rhizosphere microbial community.

### 3.5 Quorum-sensing governs the antagonism against bacterial and fungal pathogens

**Figure 6:**
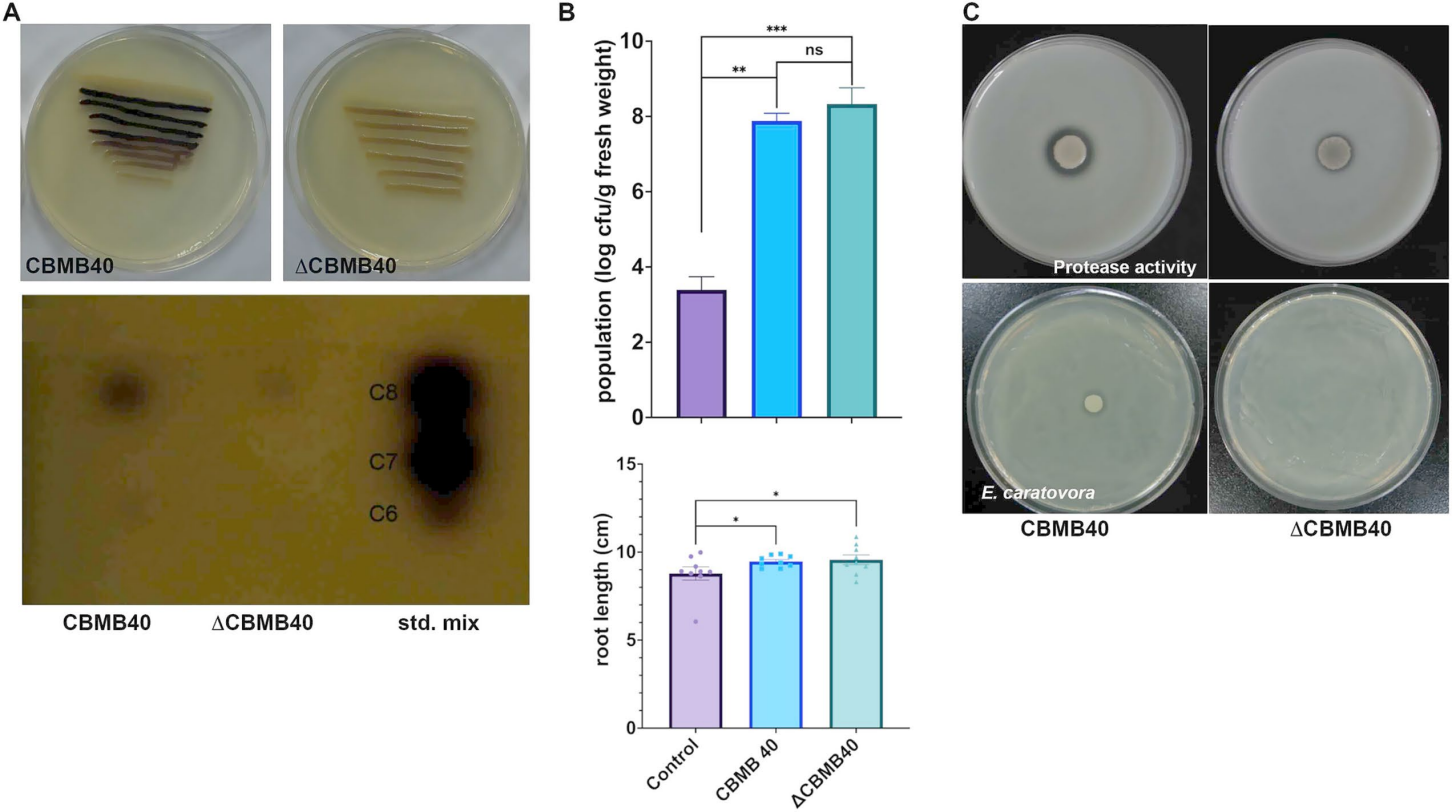
Characterization of the QS mutant (ΔCBMB40) of *B. vietnamiensis* CBMB40. **(A)** AHL production by the wild and mutant strains of CBMB40 in plate assay and TLC. The mutant strain (∆CBMB40) neither induced violacein production in *C. violaceum* CV026 in a simple plate assay nor produced any compounds when the culture supernatant extracts were separated by TLC. **(B)** ∆CBMB40 was not impaired in its colonizing ability and was able to promote the root length in inoculated plants. Each column represents the mean ± standard error of three replications. The treatments are significantly different from the control, while there is no significant difference between ∆CBMB40 and wild-type CBMB40 (* *p* < 0.05, ** *p* < 0.01, *** *p* < 0.001). **(C)** The protease activity and antagonism against the soft rot pathogen *E. caratovora* subsp. *caratovora* by wild-type CBMB40 and ΔCBMB40. The clearance zone indicates the enzyme activity (top), while the pathogen lawn growth is arrested by CBMB40, but it is not seen in ΔCBMB40.

An AHL-deficient mutant of *B. vietnamiensis* CBMB40, designated ∆CBMB40, was generated via random transposon mutagenesis as described in the Methods section. The mutant failed to induce violacein production in *C. violaceum* CV026, suggesting a lack of AHL production. TLC of concentrated culture supernatants further confirmed the absence of detectable AHLs in ∆CBMB40, while the wild-type strain produced C_6_-HSL and C_8_-HSL (Figure 6A). ∆CBMB40 also showed reduced overall growth and a prolonged logarithmic phase compared to the wild-type CBMB40 (data not shown). Despite the impaired AHL production, rhizosphere colonization by ∆CBMB40 on red pepper seedlings remained comparable to that of the wild-type strain over a 15-day period under gnotobiotic conditions. Root length measurements showed no significant differences between plants inoculated with either the mutant or the wild-type strain, indicating that AHL deficiency did not impact the colonization and root growth-promoting ability of CBMB40 under sterile conditions (Figure 6B).

Quorum-sensing in various *Burkholderia* spp. has been implicated in regulating multiple phenotypes, including siderophore production, protease and lipase activity, and antimicrobial properties, with considerable differences between clinical, environmental, and plant-associated species (Lewenza et al., 1999; McKenney et al., 1995; Jun-Ho et al., 2001; Zhou et al., 2003). *B. vietnamiensis* CBMB40 exhibited protease activity and antagonism against *Erwinia carotovora* subsp. *carotovora,* but did not produce siderophores (Poonguzhali et al., 2007). In contrast, the ∆CBMB40 mutant showed reduced, but not completely abolished, protease activity and failed to inhibit the growth of *E. carotovora* (Figure 6C).

**Figure 7:**
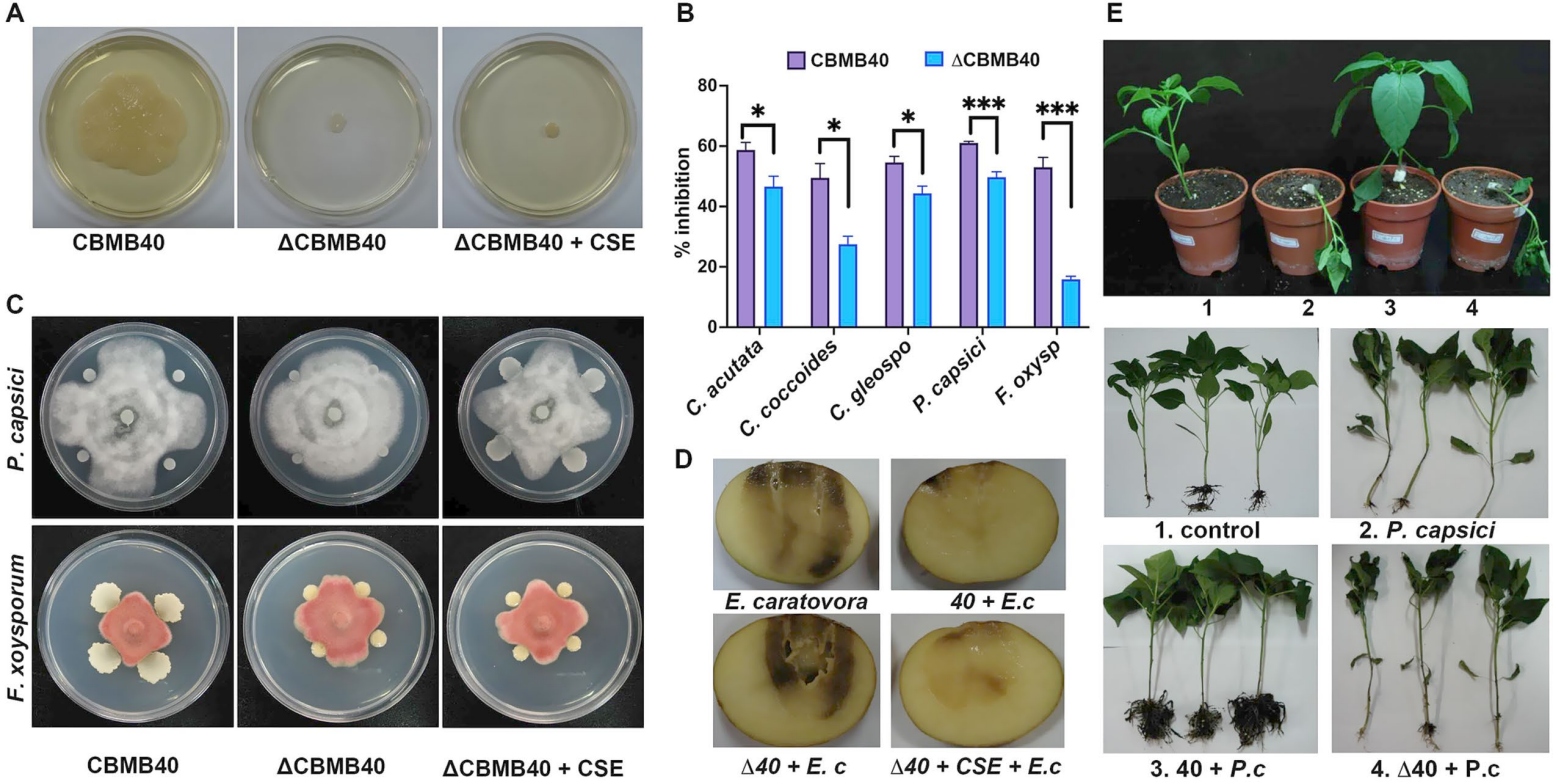
Loss of QS affects the biocontrol activity of *B. vietnamiensis* CBMB40. **(A)** Swarming motility in agar plates is lost in the QS mutant ΔCBMB40 and addition of the culture supernatant extract (CSE) is not able to restore the motility in the mutant. **(B)** Antagonism against several plant pathogens in the WT and mutant is shown as the percentage inhibition in growth compared to the control. Each column represents the mean ± standard error (SE) of three replications. ∆CBMB40 is compromised in the antagonism against the tested fungal pathogens compared to wild-type CBMB40 (* *p* < 0.05, ** *p* < 0.01, *** *p* < 0.001). **(C)** Addition of CSE of the wild-type to ∆CBMB40 could restore the antagonistic potential against fungal pathogens. Only plates showing the growth of *P. capsici* KACC 40157 and *F. oxysporum* KACC 40032 are shown, while the remaining fungal pathogens tested are shown in Supplementary Figure S4. **(D)** *In vitro* potato tuber maceration assay to show the antagonism against *E. carotovora* subsp. *caratovora*. The complete set of treatments is shown in Supplementary Figure S4. The addition of CSE to the mutant ∆CBMB40 showed reduced rot symptoms comparable to WT CBMB40. **(E)** Plant experiments to show that ∆CBMB40 is unable to control the soft rot pathogen *Phytopthora capsici* KACC 40157 in red pepper. Set of plants showing the wilting symptoms in *P. capsici* KACC 40157 treated plants. Individual plants showing the root infection and collar rot symptoms caused by *P. capsici* KACC 40157 are shown. Inoculation of CBMB40 had reduced the symptoms in pathogen-inoculated plants, while ∆CBMB40 failed to confer protection and displayed symptoms severity comparable to plants infected with the pathogen alone.

Furthermore, ∆CBMB40 showed markedly reduced *in vitro* antifungal activity against several plant pathogenic fungi tested and completely lacked swarming motility on agar plates (Figures 7A,B). To determine whether the observed phenotypic changes were directly associated with the absence of AHLs, AHL-rich extracts from the wild-type strain were added to the culture supernatant of ∆CBMB40 to check those AHL-dependent attributes. AHL extract supplementation failed to restore swarming motility in the mutant; however, it restored the antifungal activity in plate assays against most of the tested fungal pathogens (Figure 7C; Supplementary Figure S4).

### 3.6 Loss of QS reduces the suppression of bacterial and fungal rot pathogens by *Burkholderia vietnamiensis* CBMB40

The antagonistic activity of ∆CBMB40 was evaluated against both bacterial and fungal plant pathogens using *in vitro* and *in vivo* assays. In potato tuber maceration assays, the antagonistic effect of CBMB40 and its mutant ∆CBMB40 was assessed against the soft rot pathogen *E. carotovora* subsp. *carotovora*. Soft rot symptoms were scored using the scale described by Bartz (1999): no rotting (−), restricted rot <1 cm (+), small active rot 1–2 cm (++), and highly active rot >2 cm (+++). Tubers inoculated solely with CBMB40, ∆CBMB40, or with the AHL extract from CBMB40 exhibited no visible rotting symptoms. In contrast, tubers inoculated with *E. carotovora* alone developed severe maceration, scoring as highly active rot (+++). Inoculation of CBMB40 with the pathogen reduced the severity of rot, typically resulting in restricted or small active rot. However, co-inoculation of ∆CBMB40 with *E. carotovora* failed to limit soft rot development, resembling the severe symptoms caused by the pathogen alone. Notably, when AHL extracts from CBMB40 culture supernatant were supplemented to ∆CBMB40 during co-inoculation with the pathogen, the extent of tissue maceration was significantly reduced, indicating that QS signaling restored antagonistic activity (Figure 7D; Supplementary Figure S4).

Complementary pot culture experiments were conducted in red pepper plants to evaluate the antagonistic efficacy of ∆CBMB40 against the *Oomycete* pathogen *Phytophthora capsici* KACC 40157. Plants inoculated with either wild-type CBMB40 or ∆CBMB40 appeared healthy, with broader leaves and no symptoms at the early vegetative stage, similar to uninoculated controls (Figure 7E; Supplementary Figure S4). In contrast, plants challenged with *P. capsici* KACC 40157 via stem-wounding displayed characteristic disease symptoms, including brownish stem discoloration that rapidly progressed upwards from the inoculation site, followed by sudden wilting of the entire plant, as previously described by Hwang et al. (1996). Dark brown lesions were first observed on the stems of *P. capsici* KACC 40157-infected plants by day 3 post-inoculation, with complete plant wilting noted by day 6. Uprooted plants revealed collar rot and extensive root infection (Figure 7E). Notably, plants inoculated with CBMB40 and subsequently challenged with *P. capsici* KACC 40157 maintained higher survival rates and overall health, comparable to uninoculated controls. In contrast, ∆CBMB40-inoculated plants challenged with *P. capsici* KACC 40157 developed symptoms similar to those of pathogen-only treatment, indicating a loss of biocontrol efficacy (Figure 7; Supplementary Figure 4). These results suggest that while ∆CBMB40 retains plant growth-promoting traits, it fails to suppress *P. capsici* KACC 40157 infection, implying that QS mutation compromises disease suppression mechanisms.

Collectively, these findings provide preliminary but compelling evidence that QS in *B. vietnamiensis*, likely through regulation of antibiotic production, is critical for its antagonistic activity against plant pathogens.

## 4 Discussion

### 4.1 BviI/R and CepI/R QS circuits in plant-associated *Burkholderia vietnamiensis*

Members of *B. vietnamiensis*, a member of Bcc display both CepIR, the conserved QS system, and an additional BviIR QS circuit that primarily produces C_10_-HSL, the most abundant AHL in this species (Lutter et al., 2001; Gotschlich et al., 2001; Venturi et al., 2004). Although QS has been extensively studied across the Bcc, to the best of our knowledge, no recent studies have focused specifically on QS regulation or its functional role in *B. vietnamiensis*, apart from a few reports published nearly two decades ago, as cited herein. In the present study, plant-associated *B. vietnamiensis* strains CBMB40, TVV75^T^, and SXo-702 were found to produce indistinguishable suites of AHLs, comprising C_6_-, C_8_-, C_10_-, and C_12_-HSLs. Among these, C_10_-HSL was the most abundant, followed by lower levels of C_8_- and C_12_-HSLs, whereas C_6_-HSL was detected only in trace amounts and was undetectable by GC–MS (Figure 1). While previous studies have reported distinct AHL profiles between clinical and environmental *B. vietnamiensis* isolates (Gotschlich et al., 2001; Malott and Sokol, 2007), a definitive correlation between strain origin and AHL production patterns across the Bcc has yet to be established. Moreover, the role of QS in niche adaptation, such as colonization of the rhizosphere, remains poorly understood. Our findings further demonstrate that the AHL by *B. vietnamiensis* CBMB40 varied under different growth conditions and carbon sources (Figure 3), suggesting that its QS is responsive to environmental cues indicating a potential role for QS in ecological adaptation, possibly enabling fine-tuned responses to specific niches.

PCR amplifications with specific primers for *bviI/R* and *cepI/R* genes and the amino acid sequence analysis of the sequenced products clearly show that the CepIR and BviIR present in rhizosphere isolates are identical to those of *B. vietnamiensis* described so far (Figure 2). Investigation by Conway and Greenberg (2002) implied BviI/R as the principal system in *B. vietnamiensis* G4 that accounts for all AHLs produced, while Malott and Sokol (2007) revealed CepI/R as the principal system essential for the expression of *bviI*. Through employing QS mutants, the authors showed that *bviI* is responsible for the production of C_10_-HSL while *cepI* produces C_6_- and C_8_-HSLs. Results of the present study are in line with this context, based on the expression of *bviI* and *cepI* genes of strain CBMB40 in an alternative *E. coli* host, revealing that these synthases together contribute to the production of C_6_- and C_8_-HSLs, while *bviI* is responsible for the production of C_10_- and C_12_-HSLs. However, it is important to note that our results are based on TLC and GC–MS analysis, which provided both qualitative and quantitative insights into AHL production in CBMB40. The expression profile of these QS genes under different environmental conditions was not assessed, and it remains to be determined whether the differential expression of these genes contributes to niche-specific adaptation. Taken together, our results provide evidence that the BviIR and CepIR systems are conserved among *B. vietnamiensis* strains regardless of their origin, and *bviI* is responsible for the production of C_10_-HSL, the most abundant and specific AHL of this species.

### 4.2 *Burkholderia vietnamiensis* CBMB40 AHLs under *in situ* conditions and cross-communication with *Pseudomonas* in the rhizosphere

Quorum-sensing signals, once considered solely microbial communication tools, are now increasingly recognized as inter-kingdom mediators that influence plant immunity and contribute to the stability of the plant holobiont. Plant responses to AHLs vary depending on the molecule type, concentration, and duration of exposure, and in some cases, can lead to the production of QS-mimic compounds that interfere with bacterial communication (Mathesius et al., 2003; Teplitski et al., 2003). In our study, *B. vietnamiensis* CBMB40-gfp exhibited a structured colonization pattern on tomato roots, forming linear arrays along the root surface, a common colonization pattern observed among known PGPR (Ramos et al., 2002; Belimov et al., 2007), that may facilitate efficient plant–microbe interactions and enhanced perception of bacterial signaling molecules by the host. Notably, tomato plants primed with AHLs from CBMB40 altered the QS-mimic compound pattern and retained bacterial AHLs in their root exudates, indicating both perception and stability of these signaling molecules within plant tissues.

The predominance of AHL-producing bacteria in the rhizosphere compared to bulk soil (Elasri et al., 2001) supports a broader ecological role for QS in the rhizosphere. Accumulating evidence further shows that QS facilitates both inter- and intra-species communication in the rhizosphere and is not limited to population-level interactions but can also occur between individual bacterial cells at spatially distinct locations on colonized plant surfaces (Pierson et al., 1998; Steidle et al., 2002; Gantner et al., 2006). Consistent with these findings, plant-based and rhizosphere assays using biosensor strains confirmed that AHLs produced by CBMB40 are biologically active *in situ*. These molecules were capable of inducing QS-regulated phenotypes in neighbouring rhizospheric bacteria, including *Pseudomonas* spp., providing direct evidence that AHLs can mediate cross-communication at the single-cell level within the rhizosphere. Together, these results highlight the role of CBMB40-derived AHLs in shaping both plant–microbe and microbe–microbe interactions, ultimately contributing to the rhizospheric competence and beneficial traits of *B. vietnamiensis* CBMB40.

### 4.3 Quorum-sensing in *Burkholderia vietnamiensis* CBMB40 plays a role in bacterial and fungal antagonism

Quorum-sensing in Bcc has been shown to regulate diverse traits, such as siderophore production, biofilm formation, swarming motility, the secretion of extracellular enzymes, and the expression of virulence factors (Venturi et al., 2004; Aguilar et al., 2003; Kim et al., 2004; Solis et al., 2006). However, in *B. vietnamiensis* G4, none of these traits, including nitrogen fixation or antibiotic production, were found to be QS-regulated (Conway and Greenberg, 2002; Malott and Sokol, 2007). This suggests that the QS regulon in *B. vietnamiensis* may differ significantly from that of *B. cepacia*. In our study, *B. vietnamiensis* CBMB40 was shown to possess several PGP traits. Inoculation with ∆CBMB40 did not affect root colonization ability or alter root elongation, indicating that QS may not be essential for these specific traits. However, since nitrogenase activity, ACC deaminase, and indole-3-acetic acid (IAA) production in ∆CBMB40 were not evaluated, while the wild-type strain possesses all of these characteristics (Poonguzhali et al., 2007), a definitive conclusion regarding the role of QS in PGP activity cannot be drawn. Notably, QS appeared to have no impact on siderophore or protease production, nor on swarming motility in CBMB40. Importantly, our cross feeding plate assays, *in vitro* potato tuber maceration assays with *E. carotovora* subsp. *carotovora,* along with plant challenge experiments, involving *P. capsici* KACC 40157 on red pepper, demonstrated that QS plays a functional role in the biocontrol activity of CBMB40. The genus *Burkholderia* is known for its metabolic versatility, biodegradation potential, and antimicrobial properties, making it a promising source of novel bioactive compounds, many of which are regulated by QS (Gonzales et al., 2024). Thus, it is plausible that QS in CBMB40 regulates the synthesis of yet unidentified bioactive molecules responsible for its pathogen suppression activity. Alternatively, the possibility of quorum-quenching, interference with the QS systems of pathogens, should not be overlooked. CBMB40-derived QS signals may interfere with or degrade QS signal molecules produced by pathogens, thereby disrupting their virulence. However, this mechanism may not fully explain CBMB40’s efficacy, as it is active against both bacterial and fungal pathogens, which likely differ in their signaling systems.

Furthermore, CBMB40 produces a broad spectrum of AHLs, including both short- and long-chain molecules, while ∆CBMB40 lacks all AHL production. The impact of AHLs on plant growth and defense is complex. Plants can interpret long-chain AHLs as warning signals, triggering systemic resistance, while exploiting short-chain AHLs to promote root growth. This duality forms a double-edged strategy that microbes can exploit during colonization (Zheng et al., 2025). AHL mixtures produced by PGPR or even individual commercial AHLs have been shown to function as priming agents, inducing resistance to a broad spectrum of pathogens across various plant species. Interestingly, priming with a mixture of AHLs tends to favor enhanced resistance over growth promotion. The molecular mechanisms underlying AHL-induced priming have been increasingly elucidated over the past decade (Shrestha and Schikora, 2020; Duan et al., 2024; Zheng et al., 2025). Taken together, and based on the above discussion, the biocontrol ability of *B. vietnamiensis* CBMB40 is likely attributed to a combination of factors, including the direct inhibitory effects of its secondary metabolites on pathogens and the induction of systemic resistance in the plant host.

Overall, our study highlights the potential of utilizing AHL-producing PGPR, such as CBMB40 as a sustainable and ecologically viable strategy to enhance plant resistance and growth. This approach offers a promising alternative to synthetic AHL priming and transgenic methods by enabling *in situ*, regulated AHL delivery along with additional plant-beneficial functions. While field-level application as PGPR presents challenges due to environmental and microbial complexities, these can be mitigated through the use of tailored microbial consortia, advanced formulation techniques (e.g., microencapsulation, nano-biofertilizers), and integrative multiomic approaches (Aloo et al., 2022). The use of PGPR as biofertilizers is well established in modern agriculture to promote plant performance and sustainability. Using naturally AHL-producing PGPR such as CBMB40 presents several advantages over AHL priming and transgenic approaches: localized, sustained AHL delivery, synergistic plant growth promotion, environmental safety, cost-effectiveness, and scalability across different crops and ecosystems. Importantly, PGPR-based methods are non-permanent and reversible, avoiding the ecological risks and public resistance associated with genetically modified crops. These attributes position PGPR-AHL strategies as a flexible and ecologically sound alternative for enhancing crop resilience and productivity in sustainable agriculture.

## 5 Conclusion

This study highlights the potential of AHL-producing PGPR, particularly *Burkholderia vietnamiensis* CBMB40, as a sustainable alternative to synthetic and transgenic strategies for enhancing plant growth and resistance. CBMB40 effectively colonized the tomato rhizosphere, produced AHLs *in situ*, and modulated both plant–microbe and microbe–microbe interactions. We identified two functional QS circuits (CepI/R and BviI/R) with distinct roles in AHL biosynthesis, underscoring the ecological relevance of quorum-sensing in rhizosphere competence and biocontrol. Compared to transgenic AHL-producing plants, natural PGPR-derived AHLs offer better biological compatibility and the added potential to disrupt pathogen signaling. These findings open new avenues for leveraging QS-active PGPR in sustainable crop protection and productivity.

## Data Availability

The datasets presented in this study can be found in online repositories. The names of the repository/repositories and accession number(s) can be found in the article/Supplementary material.
