# Supplementary material for: The plant growth-promoting Burkholderia vietnamiensis produces acyl-homoserine lactones and modulates the quorum-sensing signaling in the rhizosphere
Source: Front Microbiol. 2025 Aug 15;16:1638793. doi: 10.3389/fmicb.2025.1638793 (PMC12394537; doi:10.3389/fmicb.2025.1638793)
Supplement: Supplementary file 1 [file Data_Sheet_1.docx]

**Supplementary Material**

**The Plant Growth-Promoting *Burkholderia vietnamiensis* Produces Acyl-Homoserine Lactones and Modulate the Quorum Sensing Signaling in the Rhizosphere**

**Supplementary Table S1.** List of primers used in this study

| **Primer name** | **Sequence** | **Description** |
| --- | --- | --- |
| YL065 (F) | 5’-GCGATGTTAATGGGCAAAAA- 3’ | Forward primer for amplifying GFP in the CBMB40-*gfp* transformants |
| YL066 (R) | 5’-TCCATGCCATGTGTAATCCT-3’ | Reverse primer for amplifying GFP in the CBMB40-*gfp* transformants |
| cepI-1V | 5´-CCCTGTAAGAGTTACCAGTT-3´ | Forward primer for amplifying *cepI* in the *B*. *vietnamiensis* strains (600 bp) |
| cepI-1R | 5´-GATATCGATCCAGCACG CCACGAC-3´ | Reverse primer for amplifying *cepI* in the *B*. *vietnamiensis* strains (600 bp) |
| cepR-3V | 5´-CTGGATGGCGCACTACCAGGC-3´ | Forward primer for amplifying *cepR* in the *B*. *vietnamiensis* strains (450 bp) |
| cepR-3R | 5´-ACGTGGAAGTTGACCGTCCGC-3´ | Reverse primer for amplifying *cepR* in the *B*. *vietnamiensis* strains (450 bp) |
| BVII-1 | 5´-CGCAAAGT-ATCGGCATAAGG-3´, | Forward primer for amplifying *bviII* in the *B*. *vietnamiensis* strains (600 bp) |
| BVII2 | 5´-CTGTTCGTCGATCTCGATCCC-3´ | Reverse primer for amplifying *bviII* in the *B*. *vietnamiensis* strains (600 bp) |
| BVIR1 | 5´-GGAATTTGACGGTGCGGTCG-3´; | Forward primer for amplifying *bviR* in the *B*. *vietnamiensis* strains (471 bp) |
| BVIR2 | 5´- ATGCTGCAGTCCAACTATCC-3´ | Reverse primer for amplifying *bviR* in the *B*. *vietnamiensis* strains (471 bp) |
| BviI-F1 | 5’ACAGGATCCCATGCTGACGTTATTG-3’ | Forward primer for amplifying and sequencing *bviI synthase in B. vietnamiensis CBMB40 BamHI restriction enzyme site underlined* |
| BviI-R1 | 5’-CCCAAGCTTTCATACCGATTGCTC-3’ | Reverse primer for amplifying and sequencing *bviI synthase in B. vietnamiensis CBMB40. HindIII restriction enzyme site underlined* |
| CepI-F1 | 5’ATAGGATCCCATGCGGACCTTCGTT-3’ | Forward primer for amplifying and sequencing *cepI* synthase in *B. vietnamiensis* CBMB40 *BamHI* restriction enzyme site underlined |
| CepI-R1 | 5’-ATAAAGCTTTCAGGCGGCGATAGC-3’ | Reverse primer for amplifying and sequencing *cepI* synthase in *B. vietnamiensis* CBMB40. *HindIII* restriction enzyme site underlined |


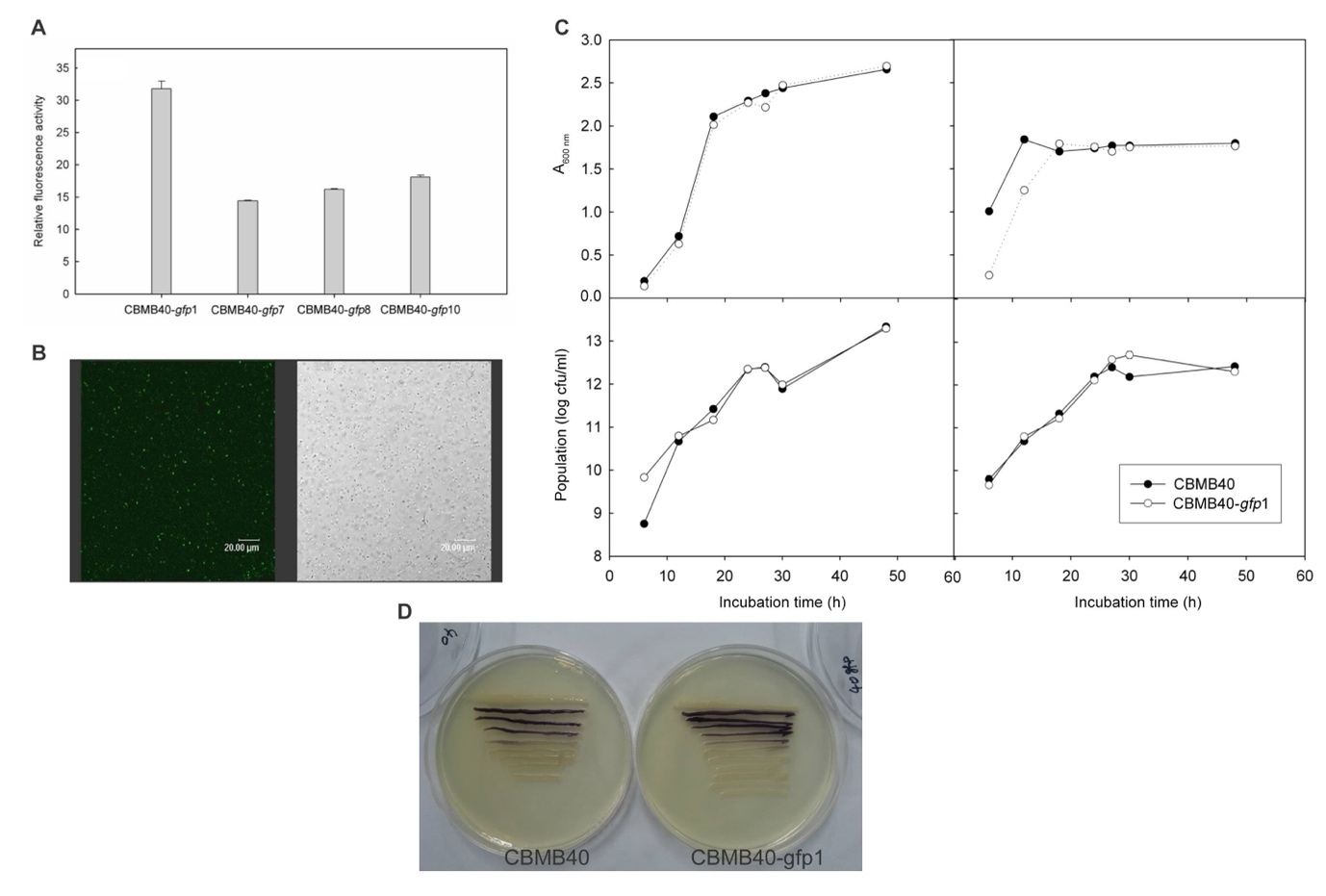


**Supplementary Figure S1.** Characterization of CBMB40-*gfp1* transformant. A. The fluorescence intensity of selected *gfp* transformants is expressed as relative fluorescence activity. B. Cells exhibiting fluorescence and phase contrast micrographs of the cells of CBMB40. C. Growth of selected *gfp* derivative of CBMB40-*gfp1* in different media. Upper panels represent the absorbance at 600 nm and the lower panels represent the population in log CFU ml^-1^ as determined by plate assays at different hours. Left - growth in KB media; Right- growth in NA+1% methanol. D. AHL production by CBMB40-*gfp1* compared with the parent strain in a cross-streak assay with *C. violaceum* CV026.


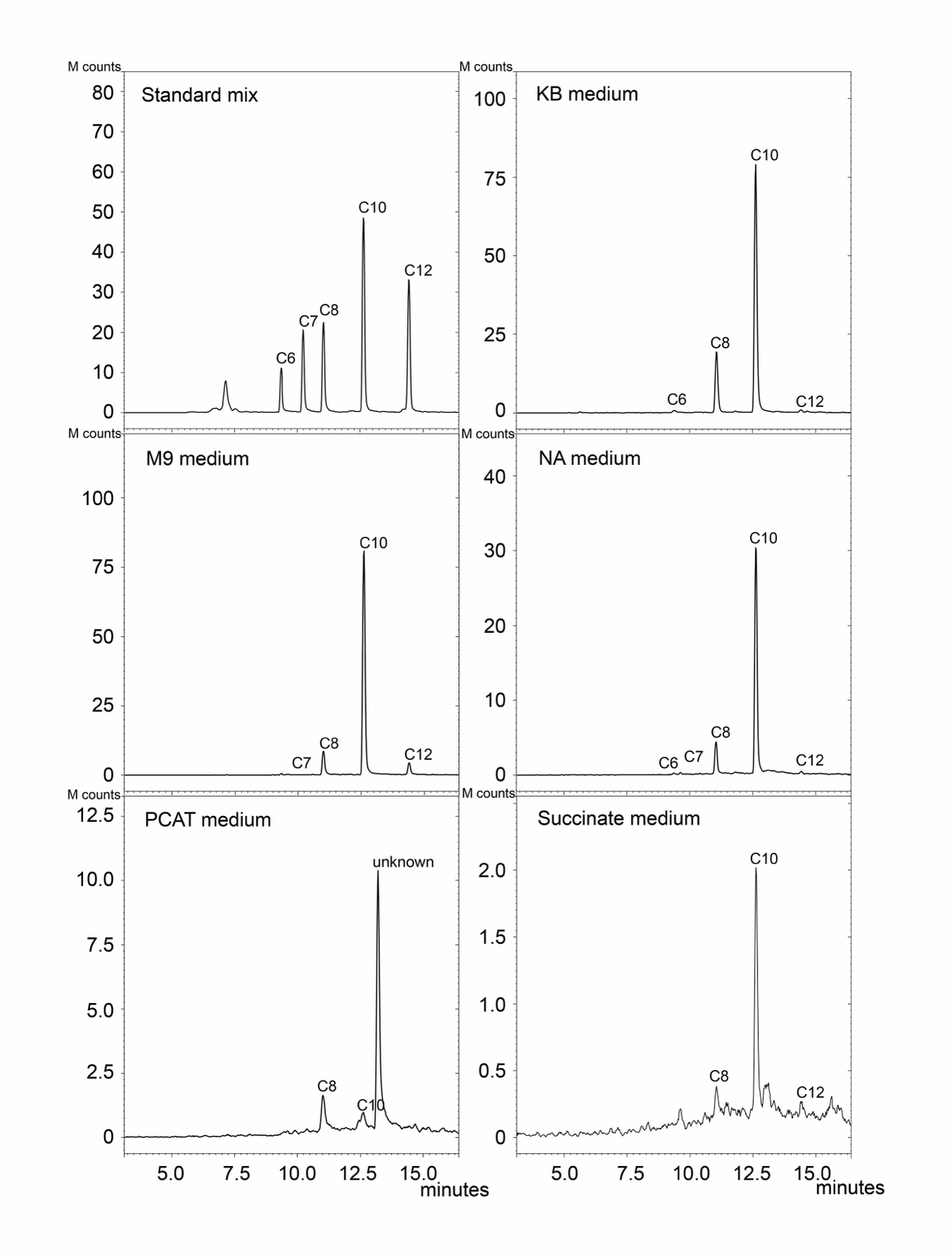


**Supplementary** **Figure S2.** Total ion chromatograms of the AHL extracts from *B. vietnamiensis* CBMB40 grown in different media. The characteristic spectrum of AHLs in each media is shown along with a mixture of standards. The media in which the bacterium was grown is mentioned.


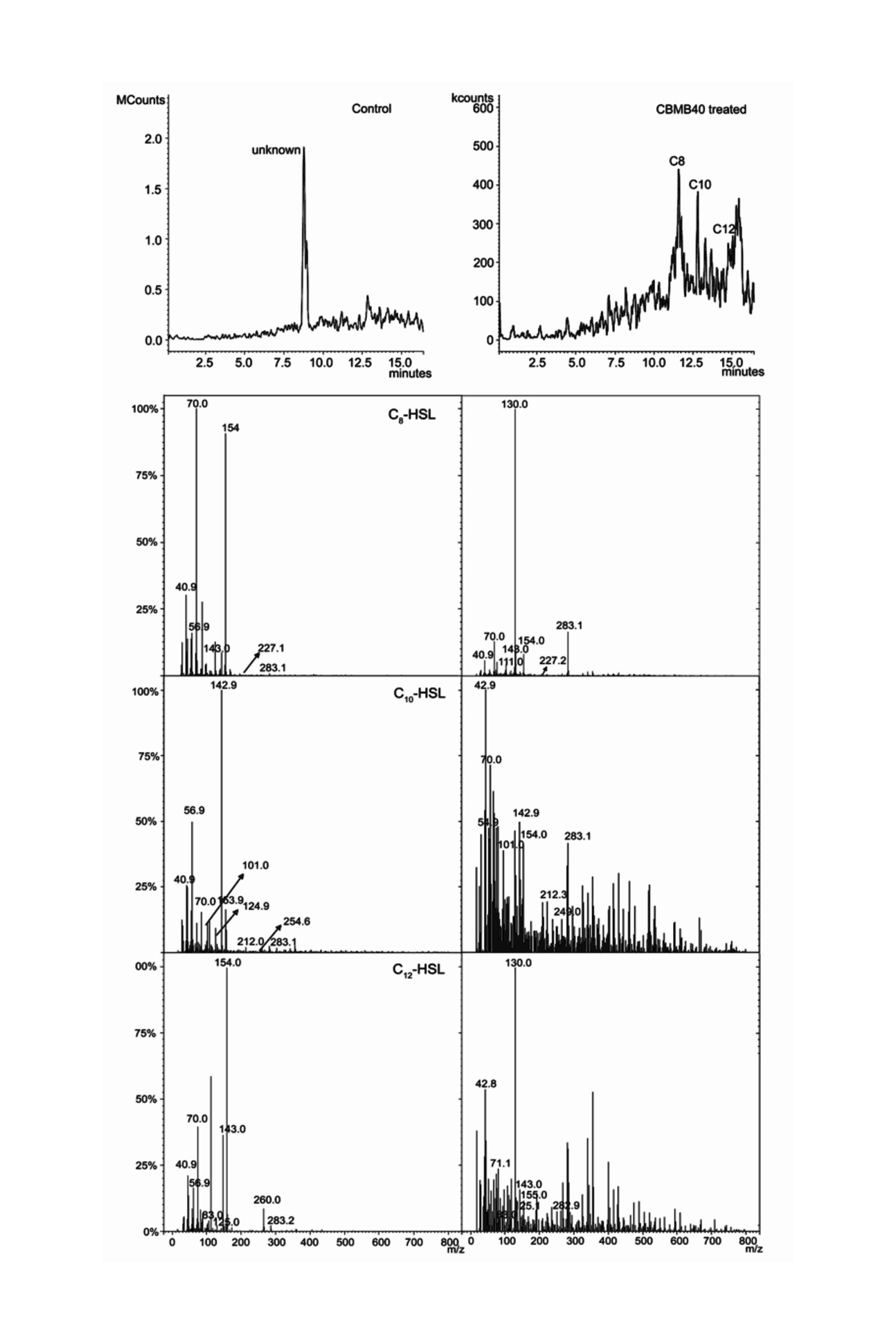


**Supplementary** **Figure S3.** GC-MS spectra of the plant extracts of tomato with and without the inoculation of *Burkholderia vietnamiensis* strain CBMB40. The upper panels represent the total ion chromatograms of the plant extracts. The lower panels show the individual spectrum of each compound (right) compared with the standard (left).

**
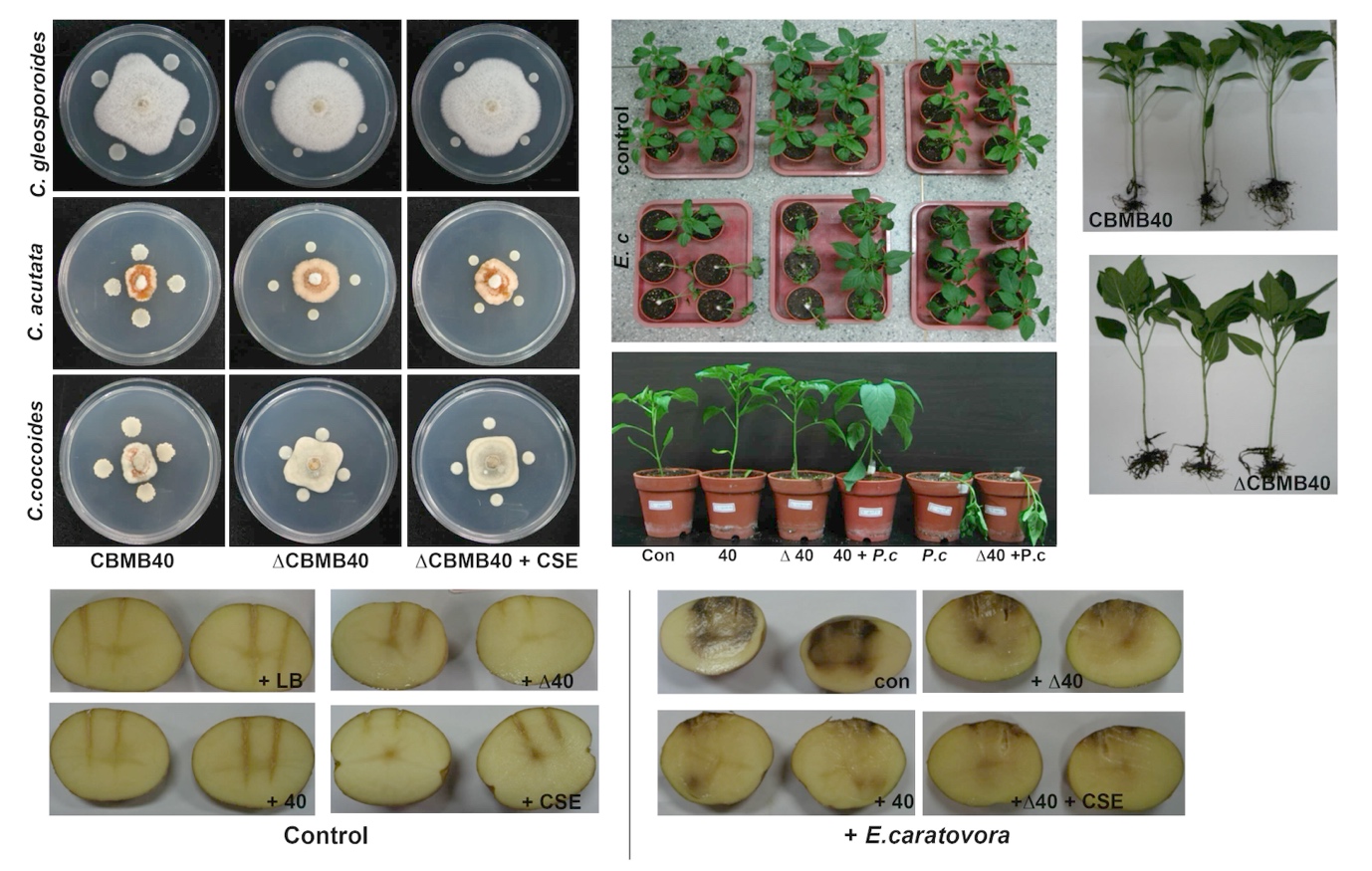
**

**Supplementary** **Figure S4.** Characterisation of ΔCBMB40. **A.** Antifungal antagonism of CBMB40 and ΔCBMB40 against the fungal pathogens mentioned. Addition of Culture supernatant extract (CSE) restored the antagonistic activity of ΔCBMB40 **B.** Plant experiments to show the biocontrol effect of CBMB40 and ΔCBMB40 on *P. capsici* KACC 40157 in red pepper. Left - The overall set up and the total treatments with representative plants for each is shown. Right- uprooted plants showing symptoms of collar rot and root infection in CBMB40 and ΔCBMB40 **C.** Potato tuber maceration assays to check antagonism against *E. caratovora subsp. caratovora* by CBMB40 and ΔCBMB40. All the treatments are shown. Control includes the treatments without pathogen infection. Right panel shows the corresponding treatments with pathogen inoculation.
